# Bioactivity and Compound Identification in Extracts from Three Australian Populations of *Portulaca oleracea*: Full NMR Structural Characterisation of Oleracein Australis 1

**DOI:** 10.3390/molecules30204147

**Published:** 2025-10-21

**Authors:** Colette Geier, Rachael Micklewright, Russell Barrow, Joanne F. Jamie, Paul D. Prenzler, Danielle Ryan, Lachlan Schwarz

**Affiliations:** 1 School of Agricultural, Environmental and Veterinary Sciences, Charles Sturt University, Locked Bag 588, Wagga, NSW 2678, Australia; cgeier@csu.edu.au (C.G.); dryan@csu.edu.au (D.R.); lschwarz@csu.edu.au (L.S.); 2 School of Natural Sciences, Macquarie University, Wallumattagal Campus, Sydney, NSW 2109, Australia; rachael.micklewright@hdr.mq.edu.au (R.M.); joanne.jamie@mq.edu.au (J.F.J.); 3 Gulbali Institute, Charles Sturt University, Locked Bag 588, Wagga, NSW 2678, Australia; rubarrow@csu.edu.au

**Keywords:** *Portulaca oleracea*, oleracein australis 1, cyclo-DOPA alkaloids, plant phenolics, antioxidant assays (TPC, TEAC, FRAP), Australian desert populations, purslane

## Abstract

Three Australian populations of *Portulaca oleracea*—Common Purslane, Omega Gold and Omega Red—were grown under identical conditions, separated into portions—leaf, bud, stem and root—and their extracts tested for total phenolic content (TPC), Trolox equivalent antioxidant capacity (TEAC), ferric-reducing antioxidant potential (FRAP), and for antioxidant activity against hydroperoxides and thiobarbituric acid reactive substances (TBARS) in a linoleic acid emulsion. Highest TPC was found in Omega Gold and Omega Red roots, with 31.1 and 36.5 mg gallic acid equivalents per gram dry weight (mg GAE/g DW), respectively, being ten times higher than for Common Purslane roots (3.1 mg GAE/g DW). Other plant portions were generally higher for Omega Gold and Omega Red, though with much less difference, i.e., <2-fold variation. Results from other antioxidant tests paralleled those of TPC. Online monitoring of antioxidant activity via post-column reaction with [2,2′-azino-bis-(3-ethyl-benzothiazoline-6-sulfonic acid)] (ABTS^●+^), revealed a peak with significant activity. Purification of the compound responsible yielded oleracein australis 1, and 1D and 2D NMR data are presented for the first time. The results of this study show that Australian populations of *P. oleracea* are high in bioactivity and may be superior to the internationally recognised medicinal plant, Common Purslane.

## 1. Introduction

*Portulaca oleracea*, also known as purslane or pigweed, is recognised globally as an important medicinal plant [1]. Endemic to most populated areas of the world, purslane is found in both temperate and tropical zones. Whilst it is believed to have originated in Southeast Asia, its presence in Australia and the Americas predates European colonisation [1]. Although now viewed as a weed within many Australian communities, purslane was historically an important food plant for the First Nations Peoples of Australia [2], and it is currently used in many other parts of the world, including Africa, Asia, South America and Europe [3].

As a medicinal plant, purslane has been shown to be efficacious, both topically and internally, treating conditions from burns to colitis, as well as complex diseases including diabetes and heart disease [4]. Its widespread use as a medicinal plant has led the United Nations Food and Agriculture Organisation to describe purslane as a global panacea [5]. This reputation has led to a significant body of natural product research to isolate the bioactive compounds responsible for the many ethnopharmacological uses of purslane [6,7]. Over 50 compounds have already been isolated from this plant, many of them previously unknown [8].

In exploring the phytochemistry of purslane in relation to non-communicable diseases, much attention has been focussed on phenolic compounds, alkaloids and polysaccharides [9]. Multiple studies have demonstrated strong bioactivity in both animals and humans, with multiple actions, including the slowing of glucose metabolism and the inhibition of pro-inflammatory pathways [10,11]. Investigation of the compounds responsible for these actions has identified a diverse range of phenolic and other compounds with a wide range of bioactivities [12].

Some of the compounds reported to be responsible for the bioactivity of purslane are unique to *P. oleracea*. These include the portulacanones, which have cytotoxic and anti-inflammatory activity [6], and the oleraceins, which generally have antioxidant activity, with one displaying neuroprotective effects [13]. Other compounds reported in purslane are flavonoids, such as kaempferol, apigenin and quercetin, which have anti-inflammatory activity [5].

Typically, studies related to the phytochemical content of purslane have been conducted on the aerial parts of the plant that are customarily used [14] and have not considered the other parts, such as the roots. By examining each plant part separately, a more complete assessment may be made of the distribution of compounds within a plant, perhaps revealing new sources of bioactivity. For example, investigation into cannabis has shown the root part to be a rich, unutilised source of medicinal compounds [15]. Thus, in this study, purslane was separated into its constituent parts—bud, leaf, stem and root—in order to investigate their contributions to its phytochemical make-up.

Australia is home to multiple populations of phenotypically unique purslane populations, several of which have proved suitable for cultivation [16]. At the time of publication, there has been no systematic botanical description of the Australian purslane phenotypes and thus the plants used in this study are referred to simply as populations rather than sub- or micro-species. The authors are also cognisant of the importance of First Nations’ intellectual property in natural products research; however, we have no way of identifying the communities from which the plant material was sourced, and have not used any Indigenous knowledge in conducting the current research.

This study investigated two Australian purslane populations and a third, which was phenotypically comparable to the Common Purslane found throughout the world. Extracts of different plant parts were evaluated for bioactivities through a series of in vitro assays. Phytochemical analyses were undertaken primarily by ultra-high performance liquid chromatography-mass spectrometry (UHPLC-MS). Nuclear magnetic resonance (NMR) spectroscopy was used to provide confirmation of structures for selected compounds characterised by MS. To the best of the authors’ knowledge this is the first report of the phytochemistry and bioactivity of Australian populations of purslane.

## 2. Results and Discussion

### 2.1. Characteristics of Australian Populations of Purslane

**Figure 1:**
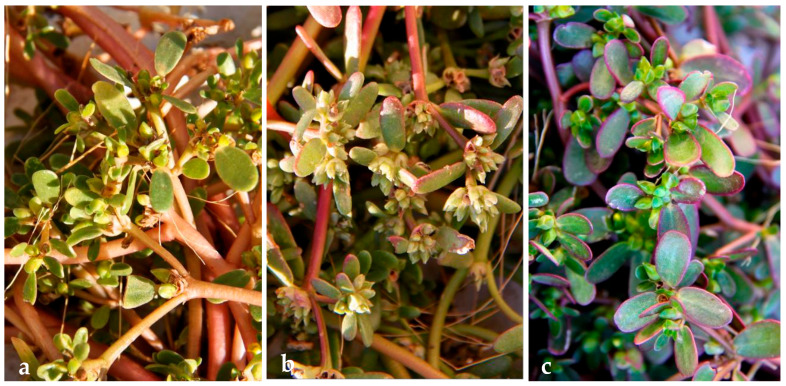
Purslane plants at time of harvest. (**a**) Common Purslane, (**b**) Omega Gold, (**c**) Omega Red.

As far as the authors are aware, no varieties or sub-species of Australian purslane have been described. Therefore, the term “populations” is used to denote the different phenotypes used in this study. Two populations were sourced from a commercial nursery and referred to in this work by their commercial names “Omega Gold” and “Omega Red” or collectively as “desert populations.” A third population was found in the Riverina, NSW, referred to here as “Common Purslane,” which is phenotypically consistent with the purslane distributed worldwide. As shown in Figure 1, the most striking visual difference among the populations was the enhanced red/purple colouration of Omega Red. In addition, Omega Gold was distinguished by its narrower leaves. As the populations were sourced from climatically distinct regions within Australia, it may be that the characteristics of the plants are a result of adaptation to different climates in those regions.

There is evidence to suggest that the phytochemistry of purslane is influenced by the conditions in which the plants are grown [17,18]. Hence in this study, the plants were grown under identical conditions for 12 weeks in order to control for growing conditions. Twelve weeks was the total time period from when the desert populations, Omega Gold and Omega Red, arrived from the supplier, were transplanted, then harvested. As Common Purslane was locally sourced, plants of the same size as Omega Gold and Omega Red were transplanted, then harvested 12 weeks later.

Given widespread interest in purslane [19], one of the primary goals of this study was to investigate whether differences in phytochemistry and bioactivity could be detected among the populations. In addition, few studies have investigated the distribution of phytochemicals and bioactivities among the plant parts [14]. Hence, on harvest, the plant material was separated into leaf, bud, stem and root.

On average, different populations produced similar amounts of biomass. The average (*n* = 5) fresh weights of whole plants were as follows: Common Purslane = 397 ± 87 g, Omega Gold = 427 ± 82 g, and Omega Red = 534 ± 225 g; and the ranges were 277–490 g, 351–530 g, and 250–878 g, respectively.

### 2.2. In Vitro Bioactivity Assays

#### 2.2.1. Total Phenolic Content (TPC)

Analysis of separated parts clearly demonstrates their different TPC (Table 1). For the desert populations, the majority of the TPC is found in the roots, suggesting that this plant part may be a useful source of bioactive compounds and worthy of further investigation (see below).

Other differences in bioactivity among the purslane populations were found in the stem, with Common Purslane having the lowest TPC, 2.40 ± 0.34 mg GAE/g DW, compared with Omega Gold, 4.19 ± 0.32 mg GAE/g DW and Omega Red, 4.65 ± 0.47 mg GAE/g DW. In the bud, Common Purslane was found to have the highest TPC, 8.97 ± 0.28 mg GAE/g DW, followed by Omega Gold, 5.47 mg GAE/g DW, then Omega Red, 3.98 ± 0.77 mg GAE/g DW. These values may be compared with respective TPC contents in other studies: 10.0 mg GAE/g DW [20], 2.4 mg GAE/g DW [21], and 5.15 mg GAE/g DW [22], which shows that the Australia populations contain comparable TPC to those from other countries.

Considering the aerial parts (bud, leaf, stem), which are the most frequently consumed plant part, and which are most considered for medicinal purposes, there is some variation among the components. For the desert populations, the buds were lower in TPC, whereas the leaf had the highest TPC. Overall, there was about a two-fold variation in TPC between the lowest part and the highest. In Common Purslane, the stem had the lowest TPC and the highest TPC occurred in the bud, with a variation of about four fold.

#### 2.2.2. Trolox Equivalent Antioxidant Capacity (TEAC) Assay

Within populations, similar patterns in TEAC values to those reported for TPC above were found among the different plant parts (Supplementary Material Table S1). For example, TEAC values for the leaf parts of Common Purslane, Omega Gold and Omega Red were similar, 92.38 ± 0.37, 96.53 ± 0.70, 98.32 ± 0.76 mmol TE/g DW, respectively. On the other hand, the TEAC value for Common Purslane roots, 37.54 ± 0.61 mmol TE/g DW, was extremely low compared with Omega Gold and Omega Red, 739.41 ± 7.14 and 880.28 ± 6.75 mmol TE/g DW, respectively.

There are only three reports of TEAC values for purslane in the literature, as far as the authors are aware. Rinaldi et al. [23], found 192 mg TE/100 g fresh weight in their study on the postharvest quality traits of purslane. Nagarani, Abirami, Nikitha, & Siddhuraju [24] reported TEAC values of 51–54 mmol TE/g extract. Neither of these studies can be compared directly to the current study, either through the use of fresh weight rather than dry weight [23] or through the reporting of values per gram of extract [24] rather than dry weight. On the other hand, Erkan [25] has reported 0.5 mmol TE/g DW for the aerial parts of purslane, which is considerably lower than the values found in the present study.

#### 2.2.3. Ferric-Reducing Antioxidant Potential (FRAP)

A strong correlation was found to exist between FRAP and ABTS results (r^2^ > 0.95) among plant parts. For plant parts, FRAP values ranged from 8.26 ± 0.01 mmol TE/g DW for the Common Purslane bud, to 204.0 ± 0.05 mmol TE/g DW for Omega Red root (see Supplementary Table S2 for all values). Comparisons with other studies in the literature are hampered by differences in the quantification of FRAP; however, some large variations have been reported. For example, Lim and Quah [26] found a five-fold variation in FRAP values (0.93–5.10 mg GAE/g fresh weight) among six varieties from Malaysia. Another study from Malaysia [27] found a three-fold difference in FRAP values for two wild-type accessions (14.0 and 41.5 mg TE/g DW).

#### 2.2.4. Antioxidant Activity in a Linoleic Acid Emulsion

According to the definition of an antioxidant—“a substance that when present in small concentrations relative to a substrate, inhibits oxidation of the substrate” [28]—in order to strictly measure antioxidant activity, a substrate is required. None of the above assays involves a substrate, and hence to further explore the antioxidant activities of purslane extracts, linoleic acid was used as a substrate in an emulsion system. The use of this system to measure antioxidant activity has been reviewed [29,30] and it meets the criteria set by Prior et al. [31] for a biologically relevant, high-throughput screening method. With the addition of an antioxidant to the linoleic acid emulsion, the inhibition of primary oxidation products, lipid peroxides, can be measured by the ferric thiocyanate assay and the inhibition of secondary oxidation products, e.g., malondialdehyde, can be measured with the TBARS assay.

Table 2 shows results for peroxide inhibition by the various plant parts of the purslane extracts. The general trends are roughly equivalent to the results for TPC and other assays, with the buds or stems displaying the least activity, and the roots the highest activity. However, there are some notable differences. In particular, Common Purslane roots showed a very high activity of 61 ± 7%, similar to Omega Gold and Omega Red, whereas in previous assays, Common Purslane roots showed greatly reduced activity compared with the desert populations. Previous reports on the comparison of lipid-based assays such as FTC with those such as TPC, have noted that antioxidant activity in the lipid-based assays can be quite different to results in other assays [32]. This has prompted researchers, such as Frankel and Meyer [28] to recommend that multi-dimensional antioxidant testing be undertaken, as has been done in the present study.

Another difference among the results from Table 1 and Table 2, is that there is relatively little difference between plant parts for the FTC results (Table 2) compared with TPC results. That is, in Table 1, Omega Gold and Omega Red roots showed considerable enhanced antioxidant activity when compared with all other plant parts, up to a 10-fold difference between the lowest and highest values. This marked difference is not found for the FTC results in Table 2, where only an approximately 2-fold difference was found. This could be due to there being different suites of compounds responsible for the two activities—TPC and FTC—and because the compounds responsible for FTC activity are more evenly distributed among the plant parts. This reinforces the need for multi-dimensional antioxidant testing of plant extracts. Different compounds can have different antioxidant activities depending on whether the testing is in an aqueous environment (TPC) or lipid environment (FTC). By utilising the different assays, a more comprehensive picture of antioxidant activity can be gauged.

Only two studies (as far as the authors are aware) have investigated lipid peroxide inhibition by purslane extracts. Lim and Quah [26] reported results from a β-carotene bleaching assay. The variation in antioxidant activity ranged from 60% to 80% across five cultivars. There was no correlation with TPC data. Aryal et al. [33] investigated eight “wild vegetables” from Nepal, including purslane. They reported a value for the FTC assay of ~60% inhibition for purslane and not a strong correlation with TPC results. The results from these studies reinforce the necessity for using a variety of assays that test different aspects of antioxidant action.

All populations of purslane were effective in inhibiting secondary oxidation products as measured by TBARS, with all plant parts from all populations having TBARS inhibition values of >90%. Previous studies have shown purslane extracts to be effective in inhibiting formation of TBARS both in vitro [25] and in vivo [34].

The results of the above assays show that, across most tests, differences in bioactivities among the populations could be detected, with Omega Red consistently showing the highest activity and Common Purslane the lowest. There is also evidence to suggest that bioactivity is not evenly distributed among the different parts of the plant. In particular, the root part seems to be an excellent source of bioactivity, which, until this study, has been largely overlooked in purslane research. Such differences in bioactivities prompted us to look at the phytochemistry of purslane extracts in more detail through ultra-high performance liquid chromatography with a variety of detection methods—diode array, online ABTS and mass spectrometry—to identify compounds that may be responsible for the observed differences.

### 2.3. Profiling of Purslane Extracts by UHPLC-DAD

Initial profiling of purslane extracts was undertaken with UHPLC-DAD with a focus on 280 nm chromatograms, which is the wavelength typically used to screen for phenolic compounds and oleraceins. Such preliminary screening may quickly reveal whether the differences in bioactivity observed can be attributed to qualitative and/or quantitative differences in compounds among populations or plant parts. However, while differences among populations (Supplementary Material Figure S1) and plant parts (Supplementary Material Figure S2) could be observed, there were no obvious features that connected the trends in bioactivities, above, with particular compounds. This was especially evident for extracts from the roots. These consistently showed high bioactivities, yet few peaks were observed in the chromatogram. It is possible that the main bioactive compounds in the root extracts elute at the void time, where there is a significant peak in the chromatogram that also has antioxidant activity (see also online ABTS chromatogram, below).

### 2.4. Online ABTS Chromatograms

**Figure 2:**
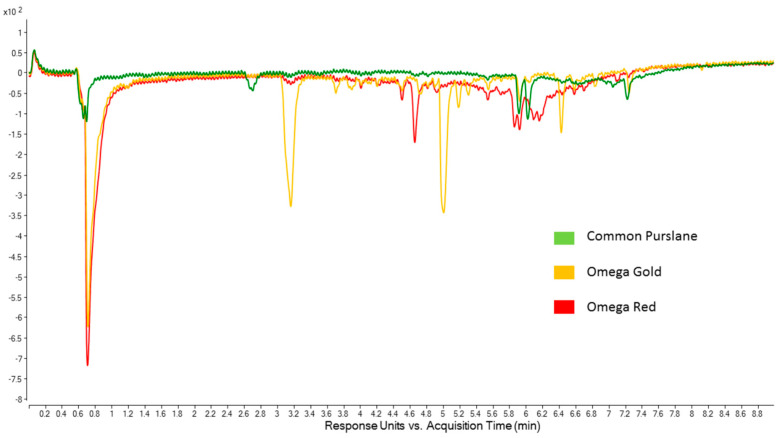
Online ABTS chromatograms (414 nm) of leaf part extracts from Common Purslane, Omega Gold and Omega Red populations.

The online UHPLC-ABTS radical scavenging assay is very useful for screening samples to identify which peaks within a DAD chromatogram may be associated with compounds with high antioxidant activity [35]. The use of a second diode array detector set at 414 nm monitors the reaction of the column eluent with the ABTS reagent. Detection of a “negative” peak is indicative of antioxidant activity, as shown in Figure 2 below.

As shown in Figure 2, qualitative and quantitative differences can be seen among the chromatograms. Comparing the results of the ABTS assay with the online ABTS chromatograms, it can be seen that Common Purslane has the fewest peaks, and that they are all of relatively low intensity, consistent with Common Purslane leaf extracts having the lowest antioxidant activity in the ABTS assay. For the desert populations, Omega Red leaf extracts had slightly higher antioxidant activity than Omega Gold leaf extracts, but a direct comparison of how that difference may be accounted for in terms of compounds (peaks in the online ABTS chromatograms) is challenging. Most of the antioxidant activity in the desert populations can be attributed to a very large peak at 0.7 min, which is near the void time. It was beyond the scope of this research to investigate these compounds further as they do not separate on a reverse phase column, but clearly their pronounced antioxidant activity warrants further study, for example via UHPLC-DAD using hydrophilic interaction liquid chromatography (HILIC) separation. The Omega Gold chromatogram is characterised by two prominent peaks at 3.2 min and 5.0 min, whereas the Omega Red chromatogram is characterised by a very broad “hump” between 3.6 min and 6.8 min, which is likely due to polymeric material [36] that effectively scavenges the ABTS radical. As well as this hump, there are some specific peaks of moderate intensity at 4.7 min and between 5.8 min and 6.2 min, which may be attributable to antioxidant compounds.

**Figure 3:**
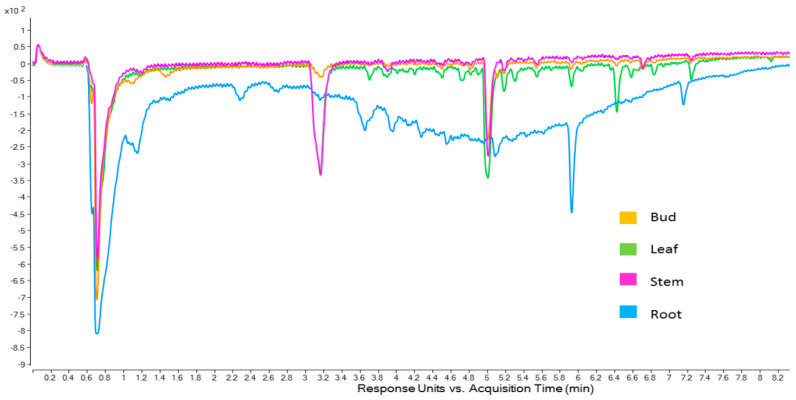
Online ABTS chromatogram (414 nm) of Omega Gold plant part extracts.

Looking within a population, information can be discerned as to the cause of differences in antioxidant activity among the various plant parts (Figure 3). The main feature that can be distinguished in Figure 3 is the very broad area of antioxidant activity in the ABTS chromatogram of the root part. Along with the early eluting peak (0.7 min), the compounds represented by these peaks make up the majority of the antioxidant activity for the roots. This was found to be, for desert populations, where the main antioxidant activity was located. Further, the broad hump is indicative of polymeric material, possibly tannins, indicating that the roots may be a good source of these antioxidants. The leaf and stem have similar profiles, although the leaf is characterised by later eluting compounds (5.0 min onwards). These later eluting compounds must impart a significant amount of antioxidant activity to the leaf extract as it had much higher activity than the stem extract (Table 1, above).

**Figure 4:**
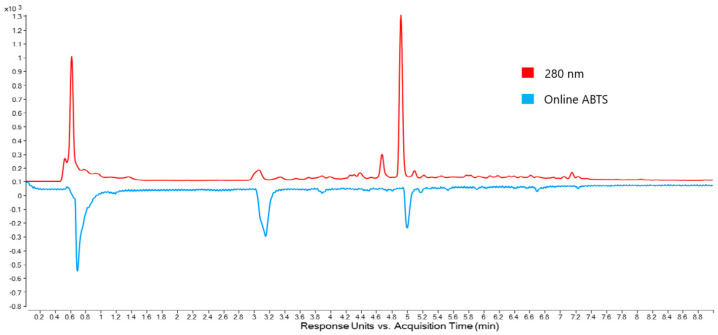
UHPLC-DAD chromatograms of Omega Gold stem extract generated at 280 nm and the corresponding ABTS chromatogram generated at 414 nm. The online ABTS chromatogram shows inverted peaks at a later retention time because the detector for the ABTS system is downstream from the DAD.

To more clearly associate antioxidant activity with compounds in Omega Gold, the stem ABTS chromatogram and the corresponding 280 nm chromatogram were plotted together (Figure 4). As can be seen in Figure 4, there is alignment (allowing for retention time offset) of the ABTS peaks and two peaks in the 280 nm chromatogram (apart from the large peak near the void time). Due to these peaks being the predominant antioxidant peaks found in purslane, further work was undertaken to assign molecular structures.

The peak at 3.2 min in the ABTS chromatogram (2.7 min, 280 nm chromatogram) showed a UV spectrum with an absorbance maximum at 280 nm indicating a simple phenol. In the LC-QTOF-MS spectrum, three peaks with *m*/*z* values of 317.1178, 339.1003 and 355.0750 may be assigned to [M + H]^+^, [M + Na]^+^, [M + K]^+^, respectively. In the negative ion mode, a peak at *m*/*z* 315.1078 may be assigned to [M − H]^−^. Accurate mass data from QTOF-MS suggest a molecular formula of C_14_H_20_O_8_. A likely candidate for this formula is hydroxytyrosol glucoside (exact mass 316.1158). Use of the LC-QTOF-MS in auto MS/MS mode revealed a key fragment of *m*/*z* 153.0153 in the negative ion mode, indicative of a hydroxytyrosol moiety following loss of glucose. This observation is consistent with the fragmentation of hydroxytyrosol 4-β-d-glucoside reported by Romero et al. [37]. However, the 4-β-d-glucoside does not have an *ortho*-diphenol group and therefore would not be expected to show strong antioxidant activity in the ABTS chromatogram. An alternate possibility is that the peak in the ABTS chromatogram is due to the 1-β-d-glucoside, with the glucose attached to the ethanol oxygen, leaving the *ortho*-diphenol available for antioxidant activity.

In an *m*/*z* 315.1078-extracted ion chromatogram (negative ion mode), two different peaks (2.2 min and 2.7 min) occurred depending on the sample extract, suggesting two isomers. The root of Omega Red was found to contain higher concentrations of the earlier eluting isomer, with all aerial parts of all populations and the root of both Omega Gold and Common Purslane containing more of the latter. The lack of an earlier eluting peak in the ABTS chromatogram indicates that this isomer may be the 4-β-d-glucoside (or 3-β-d-glucoside) of hydroxytyrosol, while the later eluting isomer may then be the 1-β-d-glucoside.

The other major peak in the ABTS chromatogram (5.1 min) was shown, via LC-QTOF-MS, to comprise [M − H]^−^ *m*/*z* 680.1829. The liquid chromatography triple quadrupole tandem mass spectrometry (LC-QQQ-MS) mass spectrum revealed fragments consistent with oleracein X, (Supplementary Material Figure S3) [38].

As far as the authors are aware, no NMR data have been published to conclusively assign the structure of oleracein X. In addition, the strong antioxidant activity displayed by this compound (Figure 4, above), warranted further investigation, including purification and multi-dimensional NMR structural characterisation.

### 2.5. Isolation and NMR Structural Characterisation of Oleracein Australis 1 and Oleracein C

**Figure 5:**
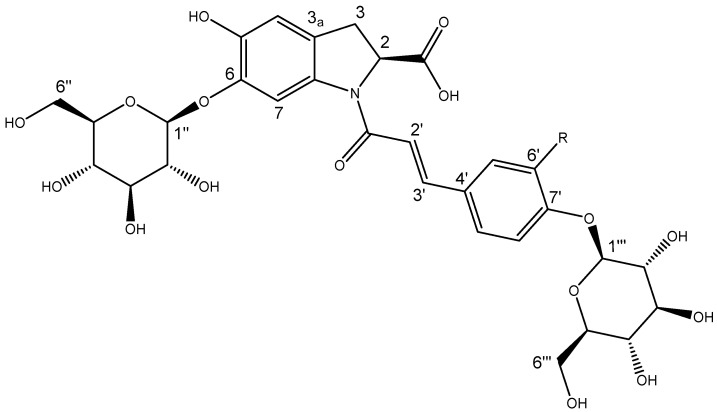
Structure of oleracein australis 1 (R = OH) and oleracein C (R = H).

Repeated reversed phase (C18) chromatography led to the isolation of two compounds with very similar ^1^H and ^13^C NMR spectra and which differed by only 16 amu—compound **1** ([M − H]^−^ *m*/*z* 664.1881) and compound **2** ([M − H]^−^ *m*/*z* 680.1829). The 1D and 2D NMR experiments of each compound showed characteristic features of oleraceins, each with a cyclo-DOPA, a cinnamoyl and two hexose moieties. The spectral data of compound **1** was in agreement with published ECD (Supplementary Material Figure S4a), MS (Supplementary Material Figure S5a,c), IR (Supplementary Material Figure S6), UV–vis (Supplementary Material Figure S7) and NMR data for oleracein C (Table 3, Figure 5 and Supplementary Material Figure S8) [39,40].

Compound **2** shared a λ_max_ of 305 nm with oleracein C but differed with a λ_max_ at 338 rather than 346 nm (Supplementary Material Figure S9). The ECD spectrum of compound **2** (Supplementary Material Figure S4b) was comparable with that of oleracein C, indicating matching stereochemistry. The ^1^H NMR spectrum of compound **2** showed an additional exchangeable proton and 1 fewer aromatic proton (Table 3). Compound **2** showed a 1,2,4-trisubstituted benzene (δ_H_ 7.19, 7.14 and 7.06) as its cinnamoyl moiety, where oleracein C showed a 1,4-disubstituted benzene seen as two doublets (δ_H_ 7.64, and 7.07) (Table 3 and Supplementary Material Figure S10). The chemical shifts of protons and carbons close to this location were also perturbed by this difference (Table 3). HMBC (Figure S10b) showed that the exchangeable proton was attached to the benzene ring of the cinnamoyl moiety and was responsible for these differences. This led to the assignment of caffeoyl as the cinnamoyl moiety of compound **2**, and the presence of a hydroxyl group in the 6′ position being the only difference in the structures. Mass spectral data were also consistent with this connectivity; compound **2** had fragment ions in negative mode at *m*/*z* 356.0778 and *m*/*z* 518.1306 (Supplementary Material Figure S5b) corresponding to oleracein C’s fragment ions *m*/*z* 340.0826 and *m*/*z* 502.1357 (Supplementary Material Figure S5a). ECD experiments revealed sequential negative and positive cotton effects at 238 and 260 nm, respectively (Figure S4b). These were in close agreement with previously reported data for (2*S*)-cyclo-DOPA and oleraceins with the same 2*S* configuration. This allowed for assignment of the stereochemistry of the cyclo-DOPA moiety as 2*S* and compound **2** was assigned as oleracein australis 1 (Figure 5), with a molecular formula C_30_H_35_NO_17_. This is the first reported isolation and structural elucidation of this molecule, hence we have given it the name “oleracein australis 1.” Oleracein australis 1 may represent the molecule detected and tentatively identified previously by MS/MS data [41,42]. An infrared spectrum of oleracein australis 1 is provided in Supplementary Material Figure S11.

**Figure 6:**
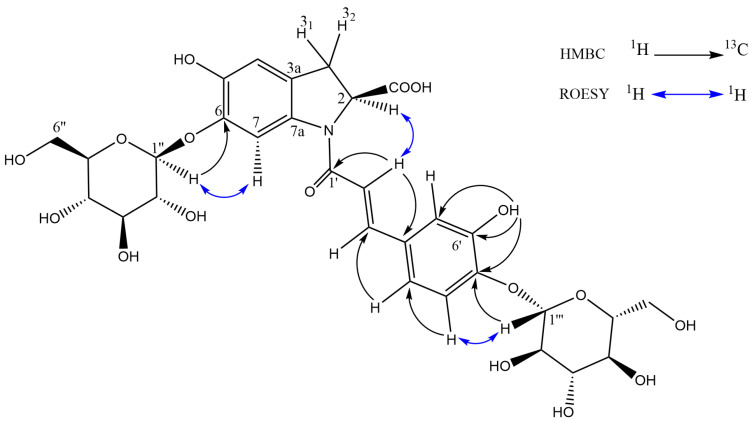
Key HMBC and ROESY correlations of oleracein australis 1.

Although the MS/MS data, above, suggest a diglucose moiety, the HMBC and ROESY data (Figure 6) conclusively prove that the two glucose units are not joined together (thus the compound could not be oleracein X), providing an important example of how MS/MS data on their own cannot be used for full structural elucidation. While the lack of an *ortho*-diphenol unit in oleracein australis 1 would seem to preclude the antioxidant activity observed in the online ABTS system, Jiao et al. [40] have shown that this moiety is not required, reporting a number of monophenolic oleraceins with antioxidant activity equivalent to ascorbic acid.

To confirm that oleracein australis 1 and oleracein C were responsible for the strong response observed in the online ABTS system, both molecules were assayed with a suite of common antioxidant assays. ABTS, DPPH, TPC and FRAP were selected to give a good spread of mechanisms and structural sensitivities. Gallic acid was selected as a standard for comparison. Both oleraceins showed substantial antioxidant activity (Table 4), with oleracein australis 1 consistently showing higher antioxidant activity than oleracein C.

### 2.6. Qualitative Profiling of Purslane Extracts

Further chemical profiling of purslane extracts was undertaken using UHPLC-QTOF-MS. Based on the proposed minimum reporting standards for chemical analysis recommended by Sumner et al. [43], the use of ‘identified’ or ‘putatively annotated’ are used for compound identification purposes. Putative annotation is the highest level of compound identification possible in the absence of pure reference standards.

For this study, 16 compounds previously reported in purslane were available as commercial standards, but only eight could be identified in the present study (Table S3). Chlorogenic acid, caffeic acid, catechin, luteolin and ferulic acid (Figure S12) were found in all parts of all populations. Apigenin, ascorbic acid, coumarin, gallic acid, kaempferol, quercetin and vanillic acid could not be detected at all. Consistent with the results from UHPLC-DAD, there were no obvious compounds that would account for the observed antioxidant activities reported above.

Extracts were also investigated for a range of bioactive compounds previously reported only in purslane and detectable at 280 nm (Table S4). Mass spectrometric and UV data consistent with portulacanones A and B (Figure S12) indicate the presence (putative annotation) of these compounds in all populations, and in most plant parts, with the aerial parts displaying higher abundances of both. Portulacanones are homoisoflavonoids, first reported in 2012 [6] in Chinese populations of purslane, and represent a new class of naturally occurring homoisoflavonoids. Portulacanone B showed strong in vitro cytotoxic activity against human cancer cell lines (NCI-H460 and SGC-7901) [6].

Another prominent class of compounds found mainly in purslane are the oleraceins. Oleraceins are a group of cyclo-dopa-based alkaloids, first reported in 2005 [39] in Chinese purslane populations. Since then, oleracein E has been isolated from other sources [44], and has received significant attention due to a promising finding in the treatment of neurodegeneration and Parkinson’s disease [45].

Oleraceins were putatively annotated mainly in the aerial parts of all purslane populations. Higher levels of oleraceins A and B were characteristic of Common Purslane. Omega Gold was characterised by high amounts of oleraceins C, D, E and H. Oleraceins K, L, O, P were detected only in Omega Red in high amounts. Omega Red was also notable for having significant quantities of oleracein E, in the root part. In other populations, the root parts only had trace to moderate amounts of oleraceins. The root part of Omega Red may be a valuable, but as yet unutilised, source of oleracein E. Oleraceins F, G, M and R could not be found in any of the current samples.

Of the oleraceins listed in Table S4, only E, K and L contain an *ortho*-diphenol moiety, which would be predicted to confer high antioxidant activity. Indeed, oleraceins K and L demonstrated twice the radical scavenging activities of ascorbic acid when tested in vitro [40]. It is significant to note that these oleraceins were only observed in desert populations, which consistently had the highest antioxidant activity. Furthermore, Omega Red had high levels of all three of these oleraceins and Omega Gold only had high levels of E, consistent with Omega Red having somewhat higher antioxidant activity than Omega Gold. This suggests the desert populations, and Omega Red in particular, may be a superior source of antioxidants than Common Purslane, which is currently cultivated as a medicinal plant.

## 3. Materials and Methods

### 3.1. Chemicals and Reagents

Reagents used without further purification were as follows: absolute ethanol, 35% hydrochloric acid (Univar, Ajax Fine Chemicals, Brisbane, Australia); *n*-hexane (HPLC grade), acetonitrile (HPLC grade), and glacial acetic acid from RCI Labscan, Bangkok, Thailand; and formic acid (98% Fluka^®^ Analytical, Adelaide, Australia). The following were all purchased from Sigma Aldrich (St. Louis, MO, USA): ammonium thiocyanate, 2,2-azino-bis(3-ethylbenzothiazoline-6-sulfonic acid) diammonium salt, chloroform, 2,2-diphenyl-1-picrylhydrazyl (DPPH), Folin–Ciocalteu (FC) reagent, 6-hydroxy-2,5,7,8-tetramethylchroman-2-carboxylic acid (Trolox), iron(II) chloride, iron(III) chloride hexahydrate, potassium persulfate, sodium carbonate, sodium hydroxide, sodium phosphate dibasic, sodium phosphate monobasic, thiobarbituric acid (TBA), 2,4,6-trichloroacetic acid, 2,4,6-tripyridyl-S-triazine (TPTZ), and Tween^®^ 20. Water used was purified by a Barnstead^TM^ GenPure^TM^ xCAD Plus Ultrapure Water Purification System (Thermo Scientific, Waltham, MA, USA).

Reference standards, used without purification, were as follows: ascorbic acid, caffeic acid, catechin hydrate, chlorogenic acid, *p*-coumaric acid, coumarin, ferulic acid, gallic acid, luteolin, quercetin, rutin, β-sitosterol, tryptophan, and vanillic acid (Sigma-Aldrich, Sydney, Australia); and apigenin and kaempferol (Extrasynthese, Genay, Cedex France).

### 3.2. Plant Material

Plants of two phenotypically unique Australian desert populations, Omega Red and Omega Gold, were purchased from Kapitany Enterprises (Melbourne, Australia). The original material used to cultivate Omega Gold was sourced from the Kimberley region of Western Australia and that of Omega Red was sourced from south of Lake Eyre in South Australia. A third population, phenotypically consistent with Common Purslane, was collected from a wild population in the Riverina region of New South Wales (34° 56′ S, 147° 46′ E). Ten plants of each population were transplanted into 1.6 m × 2 m plots, 0.6 m apart, containing 60% clay loam, 20% unwashed river sand and 20% organic material (decomposed sheep manure) located at 34° 56′ S, 147° 46′ E. The plots were in full sun and topped with white plastic sheeting to minimise moisture loss. Plants were grown for 12 weeks and irrigated with groundwater via centralised drip irrigation.

The five largest plants for each population were harvested (after 13 weeks of growth) and whole purslane plants were washed and allowed to air dry to remove residual water. Whole plants were manually separated into leaf, bud, stem, and root portions. Each plant portion was stored at −80 °C prior to freeze drying (Christ^®^ vacuum freezer, Alpha 2–4 LD plus, Osterode am Harz, Göttingen, Germany) and further storage at −80 °C. The separated plant portions were then extracted for chromatographic analysis and bioactivity assessment, as detailed below.

### 3.3. Preparation of Purslane Extracts

Freeze-dried plant material (1 g) was ground with an electric spice grinder, (Bodum, Copenhagen, Denmark) and suspended in 10 mL 50% (*v*/*v*) aqueous ethanol. The suspension was then agitated for 1 h at 21 °C before being centrifuged (5 min at 4000 rpm) (Allegra X-30R benchtop centrifuge, Beckman Coulter, Sydney, Australia). The supernatant was reserved and the procedure repeated with a further 10 mL 50% aqueous ethanol for a total volume of 20 mL of solvent per sample. The solution then underwent an *n*-hexane wash (3 × 1 mL) before being dried under a stream of nitrogen at 40 °C (Techne Dri-Block Sample concentrator 3A, Staffordshire, UK). The dried extract was stored at −18 °C until required.

### 3.4. In Vitro Bioactivity Assays

All bioactivity assays were performed in triplicate and all colourimetric testing was undertaken using a UV–vis spectrophotometer (Thermo Scientific, Helios Gamma, Waltham, MA, USA). Dried extracts (0.1 g ± 0.05 g) of each plant portion were re-constituted in 2 mL of 50% aqueous ethanol and then further diluted (1:10) in 50% aqueous ethanol.

### 3.5. Determination of Total Phenolic Content (TPC) Using Folin–Ciocalteu (FC) Reagent

The TPC assay was conducted using the method described by Obied et al. [46], with some modification. Diluted samples (100 μL) were added to H_2_O (6–7 mL) in a 10 mL volumetric flask, followed by the addition of FC reagent (500 μL) and aqueous sodium carbonate (1.5 mL, 20% *w*/*v*). The volume was then adjusted to 10.00 mL with H_2_O and incubated in the dark at room temperature (21 °C) for 1 h. A matrix blank was also prepared. Absorbance was measured at 760 nm. A seven-point gallic acid calibration curve was created (0–1 mg/mL) and results are expressed in relation to this as milligrams gallic acid equivalents per gram dry weight (mg GAE/g DW) of plant material.

The FC assay was also used for testing isolated compounds with modifications for a microtiter plate; 100 µL of FC reagent and 40 µL of sample solution was added. After 5 min 20% Na_2_CO_3_ solution was added, and the plate was left to sit for 20 min at room temperature. Each sample was tested in triplicate. A calibration curve was created using gallic acid as the standard for these measurements.

### 3.6. Trolox Equivalence Antioxidant Capacity (TEAC) Assay

A stock solution of the radical cation of [2,2′-azino-bis-(3-ethyl-benzothiazoline-6-sulfonic acid)] (ABTS^●+^) was prepared, using a method described previously [47], by incubating 5 mL of ABTS^●+^ (7 mM) and 88 μL of potassium persulfate (140 mM) in the dark for 12 h. The blue coloured solution of ABTS^●+^ was then diluted with H_2_O to produce a working standard with a spectrophotometric absorbance of 0.70 ± 0.05 at 734 nm.

Glass sample tubes were filled with 3 mL ABTS^●+^ working solution and 50 μL of sample before being incubated for 30 min at ambient temperature. Absorbance was recorded at 734 nm. A five-point Trolox calibration curve (0–10 μmol Trolox) was created, and results are expressed as millimoles Trolox equivalents per gram dry weight (mmol TE/g DW) of plant material.

The ABTS assay was also used for testing isolated compounds with modifications for a microtiter plate; 150 µL of ABTS solution was mixed with 25 µL of sample added in a microtiter plate, shaken and then kept at room temperature for 30 min in the dark. Each sample was tested in triplicate. A calibration curve was created using gallic acid as the standard for these measurements.

### 3.7. Ferric-Reducing Antioxidant Potential (FRAP) Capacity

The assay was conducted using methods described by Rao et al. [48], with some modifications. FRAP reagent was prepared daily with acetate buffer (300 mM, pH 3.6) using sodium hydroxide and acetic acid, 2,4,6-tripyridyl-s-triazine (TPTZ, 10 mM) acidified with HCl (40 mM) and FeCl_3_.6H_2_O (20 mM) at a ratio of 10:1:1 *v*/*v*/*v*. TPTZ solution was heated to 50 °C to enable dissolution and then combined with the iron(III) chloride solution prior to the gradual addition of the buffer whilst stirring to prevent immediate crystal formation within the reagent.

FRAP reagent (3.00 mL) was mixed with sample extracts (100 μL) and incubated at 37 °C for a period of 4 min. Absorbance was recorded at 593 nm. Results are expressed as millimoles Trolox equivalents per gram dry weight (mmol TE/g DW) of plant material, based on a five-point (0.0–2.0 mmol Trolox) calibration curve.

The FRAP assay was also used for testing isolated compounds with modifications for a microtiter plate; 150 uL of FRAP solution and 5 uL of sample (50 mg.mL^−1^) solution was shaken and left to incubate in the dark at 37 °C for 30 min in 96-well plates. Each sample was tested in triplicate. A calibration curve was created using gallic acid as the standard for these measurements.

### 3.8. DPPH Antioxidant Assay

Radical scavenging ability against DPPH^●^ was measured as described by Blois and Mansour with slight modifications and adapted to 96-well microtiter plates [49,50]. DPPH^●^ in ethanol solution (200 µL, 0.2 mM) was mixed with 50 mL of sample dissolved in water in a microtiter plate, shaken and kept at room temperature for 30 min in the dark. The absorbance was measured at 517 nm against 150 µL methanol with 50 µL of water added. Each sample was tested in triplicate. A calibration curve was created using gallic acid as the standard for these measurements.

### 3.9. Antioxidant Activity in a Linoleic Acid Emulsion

A linoleic acid nano-emulsion (oil-in-water) was prepared following the method of Ghani et al. [51], with slight modifications. Briefly, a 0.1 M phosphate buffer (pH 7.0) was prepared by mixing 19.5 mL of 0.2 M NaH_2_PO_4_ and 30.3 mL of 0.2 M Na_2_HPO_4_ with H_2_O (50 mL) and adjusting the pH to 7. Refrigerated buffer (50 mL) was mixed with 0.280 g of linoleic acid and 0.284 g of Tween^®^ 20 and homogenised (Ultra Turax homogeniser, T25 basic, Janka & Kunkel IKA Labortechnik, Rawang, Malaysia) at 19,000 rpm for 6 min. The coarse emulsion was kept on ice before and after being passed through a high-pressure pneumatic homogeniser (Emulsiflex^TM^–C5 AVESTIN Inc., Ottawa, ON, Canada) three times at 70–140’ 10^3^ kPa. The homogeniser was operated for a minimal amount of time prior to use in order to minimise heat in the unit. The emulsion was then visually examined for signs of separation. Droplet size was recorded using a dynamic light scattering Zetasizer (ZEN 3600, NanoZS, Malvern Panalytical Ltd., Malvern, UK) loaded with a sample combining 50 μL of emulsion with 4.95 mL of H_2_O. Droplet size was measured before and after homogenisation with the Emulsiflex^TM^.

Tests were performed in 50 mL centrifuge tubes containing the linoleic acid emulsion (2.5 mL), diluted sample (0.5 mL) and 0.1 M phosphate buffer (2 mL), combined in that order. Tubes were incubated in a 37 °C water bath with unsealed caps for 20 h before being subjected to the ferric thiocyanate (FTC) assay, measuring formation of hydroperoxides, and the thiobarbituric acid reactive substances (TBARS) assay, measuring secondary oxidation products as TBARS.

The FTC assay was conducted to measure peroxide formation using the method described by Ghani et al. [51], with some modifications. Samples were prepared in volumetric flasks (10 mL) containing H_2_O (5 mL) followed by the addition of 30% (*w*/*v*) ammonium thiocyanate (175 μL), nano-emulsion sample solution (175 μL) and 200 mM aqueous iron(II) chloride in 3.2% *v*/*v* hydrochloric acid. Samples were then made up to 10 mL with H_2_O and an incubation period of three min, at ambient temperature, was measured from the point at which the iron(II) chloride was added. Absorbance at 500 nm was recorded against a blank reference sample containing no emulsion solution. Runs of no more than five samples were performed (and staggered) to facilitate consistent incubation times. Antioxidant activity was calculated as % inhibition of peroxides (Equation (1)).

% inhibition of peroxides = [(AO − AS)/AO] × 100%(1)

where AO = absorbance of the control and AS = absorbance in the presence of an antioxidant.

Results are reported as percentage inhibition (rather than calculated against a standard curve) as is consistent with other studies of lipid peroxidation inhibition [52].

The TBARS assay was performed using the method described previously by Ghani et al. [51], with adjustments. A working solution of trichloroacetic acid (TCA) and thiobarbituric acid (TBA) was prepared by dissolving TBA (20 mM) in 15% (*v*/*v*) aqueous TCA. Pyrex centrifuge tubes (10 mL) were loaded with working TBA-TCA solution (2 mL) to which the nano-emulsion sample solution (1 mL) was added before being incubated at 80 °C for 30 min. Samples were then cooled in an ice bath before the addition of chloroform (2 mL), after which they were centrifuged (Beckman Coulter Allegra X-30R, Sydney, Australia) at 2000 rpm for 15 min. The supernatant was pipetted into a quartz cuvette and measured at 532 nm against a sample blank prepared without emulsion sample solution. The % inhibition of TBARS formation was calculated as per the FTC assay (Equation (1)).

### 3.10. Isolation of Oleracein Australis 1

An amount of 100 g of freeze-dried aerial parts of *Portulaca oleracea* Omega Gold were ground into a fine powder with a blender (Blend-X Pro 1400 W blender Kenwood, Nakhon Ratchasima, Thailand) and extracted with 70% aqueous ethanol (600 mL × 30 min × 3) with sonication [WUC-D03H ultrasonic bath (Daihan Scientific, Wonju, Korea, 120 W, 40 KHz and 40 °C)]. The ethanol was removed under reduced pressure at 40 °C and the water was removed via freeze drying at −80 °C and 0.06 mBar, yielding 23 g of an orange-brown semi-crystalline material. This material was crushed into a fine powder, suspended in 115 mL of *n*-hexane and 200 mL of water, sonicated for 30 s at room temperature, and left to sit for 20 min. The two liquid phases were separated, and the aqueous partition was extracted with 2 × 115 mL of *n*-hexane. The aqueous partition was freeze dried to yield 22 g of a red-orange solid. An amount of 18.00 g of the water partition was loaded onto a C18 column (70 × 70 mm × 40–63 μm, 188.0 g), and five fractions were collected under pressure from N_2_: fraction 1, 1.2 L of H_2_O; fraction 2, 650 mL of 40:60 EtOH:H_2_O; fraction 3, 1 L of 80:20 EtOH:H_2_O; fraction 4, 300 mL of EtOH; and fraction 5, 700 mL of EtOH. Fraction 2 yielded 1.14 g of red-brown solid. Fraction 2 (500 mg) was further fractionated by preparative HPLC [Shimadzu 20A system (Shimadzu, Kyoto, Japan) equipped with a Phenomenex Synergi^TM^ 10 µm Hydro-RP column (250 × 21.20 mm) (Phenomenex, Sydney, Australia) in isocratic mode (85% of 99.95% water with 0.05% trifluoracetic acid:15% of 99.95% acetonitrile with 0.05% trifluoracetic acid at 20 mL min^−1^) with monitoring at 340 nm]. Fractions eluting from 5–7 min were combined to yield 91 mg of a tan-orange semi-crystalline solid. An amount of 87 mg of this solid was further purified by prep-HPLC in isocratic mode (80% of 99.95% water with 0.05% trifluoracetic acid and 20% of 99.95% acetonitrile with 0.05% trifluoracetic acid at 20 mL min^−1^). Fractions from 45 to 77 min were combined to give 33.7 mg of orange solid. Final purification of 10.0 mg of the above sample was performed using semi-preparative HPLC (two C18 250 × 10.00 mm × 5 μm in tandem) in isocratic mode using 17.5% methanol, 0.1% trifluoracetic acid and 82.4% water. This afforded oleracein australis 1 at 103.5 min (3.1 mg) and oleracein C at 112.0 min (5.7 mg) (Figure 5).

Oleracein australis 1: amorphous yellow powder; C_30_H_35_NO_17_, ${\left[\alpha \right]}_{D}^{25}$ = −86.0 (*c* 0.2, H_2_O); UV-Vis (H_2_O): λ_max_ 305 and 338 nm; IR (KBr) *v*_max_: 3306, 2934, 1673, 1651, 1556, 1493, 1454, 1327, 1281, 1196, 1139, 1073, 1038, 1009, 848, 799 and 724; negative high resolution electrospray ionization mass spectrum (HRES ESI) MS *m*/*z* 680.1832 [M − H]^−^ (calc. for C_30_H_34_NO_17_: 680.1827), 518.1307, 356.0779, 246.0408, 474.1407, 161.0238, 391.7586 and 97.0853; positive HRES ESI MS *m*/*z* 682.1977 [M + H]^+^ (calc. for C_30_H_36_NO_17_: 682.1983), 163.0391, 520.1454, 325.0921, 358.0927, 307.0815, 196.0606, 487.1456, 127.0391, 358.1124 and 435.7098. Nuclear magnetic resonance (NMR) spectra were acquired using a 600‘54 Ascend NMR spectrometer (Bruker, Karlsruhe, Germany) for ^1^H (600 MHz), ^13^C (150 MHz), HMBC, HSQC, COSY and ROESY spectra, and a 500‘54 Ascend NMR spectrometer (Bruker, Billerica, MA, USA) for ^13^C (125 MHz) spectra. Infrared spectra were acquired using a Nicolet™ iS5 FT-IR spectrometer (Thermo Fisher Scientific, Madison, WI, USA). High resolution electrospray ionisation tandem mass spectrometry (HR-ESI-MS/MS) spectra were obtained on a Thermo Scientific Q Exactive mass spectrometer (Thermo Fisher Scientific, Germering, Germany). Ultraviolet−visible (UV−vis) spectra were obtained on a Varian Cary 1 Bio UV–Visible Spectrophotometer (Agilent, Melbourne, Australia). Optical rotation measurement was conducted on a JASCO P-1010 polarimeter (JASCO, Tokyo, Japan).

Oleracein C: amorphous yellow powder; C_30_H_35_NO_16_, ${\left[\alpha \right]}_{D}^{25}$ = −114.1 (*c* 0.2, H_2_O); UV-Vis (H_2_O): λ_max_ 305 and 346 nm; IR (KBr) *v*_max_: 3334, 2929, 1721, 1694, 1640, 1604, 1581, 1508, 1491, 1429, 1334, 1271, 1177, 1071, 1039, 1014, 922, 862, 824, 744 and 715; negative HRES ESI MS *m*/*z* 664.1881 [M − H]^−^ (calc. for C_30_H_34_NO_16_: 664.1878), 502.1357, 340.0826, 246.0408, 474.1407, 161.0238, 391.7586 and 97.0853; positive HRES ESI MS *m*/*z* 666.2015 [M + H]^+^ (calc. for C_30_H_36_NO_16_: 666.2034), 147.0439, 309.0966, 504.1498, 291.0859, 342.0969, 307.0815, 196.0603, 165.0545, 127.0389, and 85.0287. Nuclear magnetic resonance (NMR) spectra were acquired using a 600‘54 Ascend NMR spectrometer (Bruker, Karlsruhe, Germany) for ^1^H (600 MHz), ^13^C (150 MHz), HMBC, HSQC, COSY and ROESY spectra, and a 500‘54 Ascend NMR spectrometer (Bruker, Karlsruhe, Germany) for ^13^C (125 MHz) spectra. Infrared spectra were acquired using a Nicolet™ iS5 FT-IR spectrometer (Thermo Fisher Scientific, Madison, WI, USA). High resolution electrospray ionisation tandem mass spectrometry (HR-ESI-MS/MS) spectra were obtained via a Thermo Scientific Q Exactive mass spectrometer (Thermo Fisher Scientific, Germering, Germany). Ultraviolet−visible (UV−vis) spectra were obtained on a Varian Cary 1 Bio UV–Visible Spectrophotometer (Agilent, Melbourne, Australia). Optical rotation measurement was conducted on a JASCO P-1010 polarimeter (JASCO, Tokyo, Japan).

### 3.11. Chromatographic Analyses of Plant Portions

Dried sample extracts were reconstituted with 50% (*v*/*v*) aqueous ethanol (2 mL), and prepared standards (1 mg/mL). All samples were filtered (nylon syringe filter, 0.22 μm, Phenomenex, Sydney, Australia) and diluted as required to prevent detector overload whilst ensuring maximum peak detection. Two solutions of mixed standards were prepared (0.1 mg/mL) and injected after every five plant samples for quality control purposes and to ensure consistent retention times and detector response [solution 1. ascorbic acid, caffeic acid, coumarin, *p*-coumaric acid, ferulic acid, gallic acid, quercetin, β-sitosterol, tryptophan, vanillin; solution 2. apigenin, catechin, kaempferol, luteolin, rutin, tyrosine]. Similarly, 50% (*v*/*v*) aqueous ethanol blanks were also run intermittently to check for carry over from the column.

### 3.12. Ultra-High Performance Liquid Chromatography-Diode Array (UHPLC-DAD) with Online ABTS Detection

UHPLC-DAD was performed using an Agilent 1290 Infinity LC System (Santa Clara, CA, USA), consisting of a G4024A quaternary pump, G422A sampler, G1316C thermostatted column compartment, coupled with a G4212B DAD. A Kinetex^®^ (Phenomenex, Sydney, Australia) EVO C18 column (100 × 2.1 mm × 1.7 μm) was used, fitted with a Phenomenex^®^ Securityguard^TM^ ULTRA guard column (2.1 mm × 1.7 μm). The system was run with Agilent MassHunter data acquisition software (version 12.2) and Agilent MassHunter Qualitative Analysis (version B.07.00) was used for data analyses. The UHPLC procedure employed was based on the methodology described by Ying et al. (2018) [53], with some modifications. The mobile phases were water (solvent A) and acetonitrile (solvent B), both acidified with 0.1% (*v*/*v*) formic acid. Separation was achieved using a linear gradient elution wherein the mobile phase composition ranged from 2% solvent B to 100% solvent B over 20 min with a further equilibration time of five min per run. The flow rate was 0.4 mL min^−1^ with a column pressure of 400 bar. Sample injection volume was 2.00 μL and a needle wash of five seconds with 50% (*v*/*v*) ethanol was employed. Temperature parameters were not specified, leaving the runs to occur at ambient temperature (20 ± 1 °C). UV–visible spectra were recorded at 180, 230, 380, 480 and 550 nm; spectrum range was 210 to 600 nm with a step of 2 nm.

An online ABTS detector was added to the UHPLC system post-column to concurrently measure areas of antioxidant activity within sample runs. A secondary system comprised a degasser (G1379B Agilent, Santa Clara, CA, USA)) to treat the ABTS solution and a binary pump (G1312B Agilent 1260, Santa Clara, CA, USA)) to introduce ABTS solution at a flow rate of 0.35 mL min^−1^ into the UHPLC eluent line, for a combined flow rate of 0.75 mL min^−1^. The mixture then ran through a 41.5 cm mixing coil (0.127 mm internal diameter) at 37 °C to a second DAD (Varian 9050, Melbourne, Australia.) set at 414 nm. ABTS solution was prepared as described above and was protected from light by wrapping the container in aluminium foil. All UHPLC data were recorded using MassHunter acquisition software 12.2 and manipulated using MassHunter Qualitative Analysis B.07.00.

### 3.13. LC–QTOF-MS Analysis

An Agilent 1200 LC (Agilent, Santa Clara, CA, USA) coupled to an Agilent 6540 QTOF MS detector (Santa Clara, CA, USA)was used with the same column and guard column as above. The injection volume was 2 μL, and a flow rate of 0.4 mL min^−1^ was used, with a gradient as above. The QTOF MS was operated with a nebulizer gas flow of 6 L/min at 45 psi and 350 °C. Octupole radiofrequency voltage was set at 750 V with other scan source parameters, including a skimmer1 value of 65 V, fragmentor of 175 V and a nozzle voltage of 1000 V, and scan range *m*/*z* 100–1200. The system was run in both negative and positive electrospray ionisation modes. To ensure mass accuracy of recorded ions, continuous internal calibration was performed during analyses.

The mass error of the LC-QTOF-MS was calculated based on analysis of commercial standards [54] as ≤5.4 ppm, thus demonstrating the reliability of the instrument. A maximum mass error of 10 ppm, to accommodate instrument drift, and a minimum peak size of 1 × 10^4^ were required to positively characterise the presence of a compound. When searching for the presence of compounds, extracted ion chromatograms (EICs) were generated in both positive and negative ion modes, based on [M + H]^+^ and [M − H]^−^ ions, respectively. For comparison purposes, the relative abundance of compounds was based on peak heights in the EIC traces and recorded as trace ≤ 1 ×10^4^ (tr), moderate ≥ 1 × 10^5^ and ≤ 1 × 10^6^ (+), and high ≥ 1 × 10^6^ (high) (cf. [55]). In some cases, putative identification was aided by the presence of adduct ions such as [M + Na]^+^, [2M + H]^+^.

### 3.14. LC-QQQ-MS/MS Analysis

LC-QQQ-MS/MS was performed with an Agilent 1200 Series LC System combined with a 6460 Triple Quadrupole MS detector (Santa Clara, CA, USA) equipped with a Jet Stream Technology electrospray ion source in positive and negative ion modes, using the same columns, mobile phases and separation conditions as described above. The MS was operated with a sheath gas flow set at 6 L/min and 350 °C. Nebulizer gas pressure was set at 45 psi and temperature 325 °C. Capillary voltage was set at 3500 V (for both positive and negative ionisation modes) and the nozzle voltage at 500 V (both positive and negative modes). Dwell time was set at 50 ms for each selected reaction monitoring (SRM) transition, with collision energy of 40 eV.

All analyses were performed in triplicate, and one-way ANOVA and Tukey–Kramer’s post hoc test were performed in Excel (*p* < 0.05).

## 4. Conclusions

This study examined three populations of Australian purslane, two of which were phenotypically unique (Omega Gold and Omega Red) and the third physically consistent with purslane varieties and populations throughout the world (Common Purslane). Aqueous ethanol extracts of the bud, leaf, stem and root were prepared and tested in a series of assays to screen for bioactivity. The desert populations (Omega Red and Omega Gold) demonstrated the highest activity in all assays. In all tests the root part displayed the highest activity followed by the leaf, then bud and stem. Given the superior bioactivity of the root part, further work on its medicinal potential should be undertaken.

A phenolic compound, putatively annotated as hydroxytyrosol 1-β-d-glucoside glucoside, is reported in purslane for the first time. The discovery of hydroxytyrosol glucoside through the on-line ABTS system highlights the usefulness of this technique for identifying compounds with antioxidant activity and potential bioactivity. For the first time, NMR data are provided to conclusively establish the structure of oleracein australis 1.

The bioactivity tests, together with chemical profiling, suggest that Australian purslane may have potential as medicinal plants, with activities superior to the well-established Common Purslane. Furthermore, these purslane populations constitute just a small fraction of the Australian populations currently identified, and there may be others with even stronger medicinal potential.

## Figures and Tables

**Table 1:** Total phenolic content of *P. oleracea* plant parts in three Australian populations.

Plant Part	Common Purslane (mg GAE/g DW) ^1^	Omega Gold (mg GAE/g DW)	Omega Red (mg GAE/g DW)
Bud	8.97 ± 0.28 ^a,A^	5.47 ± 0.82 ^b,C^	3.89 ± 0.77 ^b,C^
Leaf	8.24 ± 0.22 ^a,A^	8.67 ± 0.41 ^a,B^	8.97 ± 0.59 ^a,B^
Stem	2.4 ± 0.34 ^b,B^	4.19 ± 0.32 ^a,C^	4.65 ± 0.47 ^a,C^
Root	3.09 ± 0.69 ^c,B^	31.13 ± 0.77 ^b,A^	36.50 ± 9.13 ^a,A^

^1^ Data are presented as means ± standard deviation. Different lowercase letters in the same row indicate significantly different (*p* < 0.05) values among populations. Different uppercase letters in the same column indicate significantly different (*p* < 0.05) values among plant parts.

**Table 2:** Percent inhibition of peroxide formation in a linoleic acid emulsion for *P. oleracea* plant parts in three Australian populations.

Plant Part	Common Purslane ^1^	Omega Gold	Omega Red
Bud	33.88 ± 0.97 ^b,C^	47.68 ± 0.86 ^a,B^	53.69 ± 8.58 ^a,B^
Leaf	57.80 ± 2.40 ^b,A,B^	58.92 ± 1.63 ^b,A,B^	67.58 ± 2.40 ^a,A^
Stem	51.03 ± 2.32 ^b,B^	61.84 ± 6.43 ^a,A^	58.83 ± 1.72 ^a,b,A,B^
Root	61.41 ± 7.03 ^a,A^	68.01 ± 7.98 ^a,A^	68.87 ± 4.89 ^a,A^

^1^ Data are presented as means ± standard deviation. Different lowercase letters in the same row indicate significantly different (*p* < 0.05) values among populations. Different uppercase letters in the same column indicate significantly different (*p* < 0.05) values among plant parts.

**Table 3:** ^1^H (600 MHz) and ^13^C (150 MHz) NMR data for oleracein australis 1 and oleracein C in d_6_-DMSO (*δ* in ppm, *J* in Hz).

	Oleracein Australis 1		Oleracein C	
Position	δC	δH (*J* in Hz)	δC	δH (*J* in Hz)
2	59.9	5.53 d ^C^ (10.8)	60.2	5.52 dd (10.8, 1.9)
2-COOH ^B^	173.4	13.20 bs	173.4	13.18
3_1_	32.1	3.15 m	32.5	3.16 m
3_2_^A^		3.51 m		3.52 m
3a	124.6	na	124.6	na
4	111.5	6.73 s	111.8	6.73 s
5	143.6	na	143.6	na
5-OH ^B^	na	8.45 bs	na	8.51 s
6	144.0	na	144.0	na
7	107.5	8.11 s	107.9	8.12 s
7a	135.3	na	135.3	na
1′	163.6	na	163.5	na
2′	117.6	6.77 d (15.2)	117.7	6.83 d (15.2)
3′	141.2	7.47 d (15.2)	141.2	7.56 d (15.2)
4′	129.6	na	128.6	na
5′	114.1	7.19 d (1.6)	129.5	7.64 d (8.7)
6′	146.8	na	116.5	7.07 d (8.7)
6′-OH ^B^	na	8.75 bs	na	na
7′	147.0	na	158.7	na
8′	115.9	7.14 d (8.5)	116.5	7.07 d (8.7)
9′	120.5	7.06 d ^C^ (8.5)	129.5	7.64 d (8.7)
1″	103.2	4.57 d (6.2)	103.6	4.61 d (7.4)
2″ ^A^	73.1	3.27 m	73.4	3.26 m
3″ ^A^	75.8	3.2–3.55 m	77.1	3.2–3.55 m
4″ ^A^	69.3	3.2–3.55 m	69.7	3.2–3.55 m
5″ ^A^	76.8	3.2–3.55 m	75.0	3.2–3.55 m
6_1_″	60.4	3.72 m	60.6	3.71 m
6_2_″ ^A^		3.48 m		3.47 m
6″-OH ^B^	na	4.47 t (5.9)	na	4.47 bs
1‴	101.4	4.75 d (7.2)	100.1	4.92 d (7.4)
2‴ ^A^	72.9	3.30 m	73.2	3.29 m
3‴ ^A^	75.6	3.2–3.55 m	77.0	3.2–3.55 m
4‴ ^A^	69.1	3.2–3.55 m	69.2	3.2–3.55 m
5‴ ^A^	77.1	3.2–3.55 m	76.6	3.2–3.55 m
6_1_‴	60.1	3.72 m	60.3	3.71 m
6_2_‴		3.61 m		3.61 m
6‴-OH ^B^	na	4.61 ^B^ t (5.9)	na	4.56 bs

^A^ Due to overlap with DMSO and H_2_O signals in ^1^H NMR spectrum, specific assignments inferred from 2D spectral data. ^B^ Proton exchangeable with D_2_O. ^C^ Long range coupling not observed due to peak broadening.

**Table 4:** Antioxidant activity of oleraceins expressed in gallic acid equivalents (mol/mol).

Antioxidant Assay	Oleracein Australis 1 ^1^	Oleracein C
ABTS	1.98 ± 0.07	1.42 ± 0.05
DPPH	1.62 ± 0.07	1.17 ± 0.04
TPC	2.08 ± 0.01	1.64 ± 0.01
FRAP	1.51 ± 0.09	1.36 ± 0.08

^1^ Data are presented as means ± standard deviation.

## Data Availability

The raw data supporting the conclusions of this article will be made available by the authors on request.

## Supplementary Materials

The following supporting information can be downloaded at: https://www.mdpi.com/article/10.3390/molecules30204147/s1, Tables of data for TEAC and FRAP antioxidant tests, compounds identified by UHPLC-QTOF-MS, and putatively identified compounds in *P. oleracea*; figures showing 1D, HSQC and HMBC NMR and mass spectra; and structures of compounds reported in *P. oleracea* and identified or putatively identified in this study.
