# Supplementary material for: Bioactivity and Compound Identification in Extracts from Three Australian Populations of Portulaca oleracea: Full NMR Structural Characterisation of Oleracein Australis 1
Source: Molecules. 2025 Oct 21;30(20):4147. doi: 10.3390/molecules30204147 (PMC12566405; doi:10.3390/molecules30204147)
Supplement: Supplementary file 1 [file molecules-30-04147-s001.zip › molecules-3882348-supplementary.pdf]

Table S1. Results for the Trolox equivalent antioxidant capacity (TEAC) assay for *Portulca oleracea* plant parts in three Australian populations.

Plant part	Common purslane[1] (mmol TE/g DW)	Omega Gold (mmol TE/g DW)	Omega Red (mmol TE/g DW)
bud	44.12 ± 0.35^{c,B}	69.84 ± 0.44^{b,B,C}	88.64 ± 0.76^{a,B}
leaf	92.38 ± 0.37^{a,A}	96.53 ± 0.70^{a,B}	98.32 ± 0.76^{a,B}
stem	26.40 ± 0.09^{b,D}	30.03 ± 0.11^{b,C}	65.79 ± 0.26^{a,b,B}
root	37.54 ± 0.61^{b,C}	739.41 ± 7.14^{a,A}	880.28 ± 6.75^{a,A}

[1] Data are presented as means ± standard deviation. Different lowercase letters in the same row indicate significantly different ($p < 0.05$) values among populations. Different uppercase letters in the same column indicate significantly different ($p < 0.05$) values among plant parts.

Table S2. Results for the ferric ion reducing antioxidant potential (FRAP) assay for *Portulaca oleracea* plant parts in three Australian populations.

Plant part	Common purslane[1] (mmol TE/g DW)	Omega Gold (mmol TE/g DW)	Omega Red (mmol TE/g DW)
bud	8.26 ± 0.01^{b,B}	6.03 ± 0.01^{b,B}	17.26 ± 0.04^{a,C}
leaf	17.20 ± 0.01^{c,B}	23.94 ± 0.03^{b,B}	31.86 ± 0.04^{a,B}
stem	11.48 ± 0.00^{c,B}	19.86 ± 0.02^{b,B}	21.98 ± 0.01^{a,B,C}
root	33.33 ± 0.06^{b,A}	180.87 ± 0.08^{a,A}	204.02 ± 0.05^{a,A}

[1] Data are presented as means ± standard deviation. Different lowercase letters in the same row indicate significantly different ($p < 0.05$) values among populations. Different uppercase letters in the same column indicate significantly different ($p < 0.05$) values among plant parts.

Table S3. Identification of compounds in *P. oleracea* extracts by liquid chromatography-quadrupole-time-of-flight mass spectrometry using commercial standards.

	Common Purslane				Omega Gold				Omega Red			
Compound	Bud	Leaf	Stem	Root	Bud	Leaf	Stem	Root	Bud	Leaf	Stem	Root
Chlorogenic acid	+*	+	+	tr	+	tr	tr	+	high	+	+	tr
Caffeic acid	+	+	+	+	+	+	+	+	+	+	+	+
Catechin	+	tr	tr	+	+	tr	tr	+	+	tr	tr	+
Ferulic acid	+	+	+	+	+	+	+	+	+	+	+	+
Luteolin	+	+	tr	+	+	tr	+	+	tr	-	-	-
p-coumaric acid	tr	tr	tr	tr	tr	tr	tr	tr	tr	tr	tr	tr
Quercetin	+	tr	tr	+	tr	tr	+	+	+	tr	+	+
Rutin	tr	tr	tr	tr	tr	tr	tr	tr	tr	tr	tr	tr

*abundance based on peak height in extracted ion chromatograms and designated: - = not detected; tr = trace ≦ 1×10^4; + = moderate ≧ 1×10^5 and ≤ 1×10^6 (+); high ≧ 1×10^6.

Table S4. Putative identification of compounds reported to be unique to *P. oleracea* by liquid chromatography-quadrupole-time-of-flight mass spectrometry.

	Common Purslane				Omega Gold				Omega Red			
Compound	Bud	Leaf	Stem	Root	Bud	Leaf	Stem	Root	Bud	Leaf	Stem	Root
Portulacanone A	+*	tr	tr	+	+	tr	+	+	+	tr	+	+
Portulacanone B	+	-	tr	+	tr	-	tr	+	+	tr	+	tr
Oleracein A	+	+	high	+	+	+	+	tr	+	+	+	tr
Oleracein B	high	high	high	+	+	+	+	tr	+	+	+	tr
Oleracein C	high	+	+	tr	high	high	high	+	+	+	+	tr
Oleracein D	high	+	+	+	+	+	high	-	+	+	+	-
Oleracein E	+	tr	tr	tr	+	tr	high	+	+	+	tr	high
Oleracein H	+	tr	+	+	high	+	high	+	+	tr	+	-
Oleracein I	high	high	+	+	tr	+	+	tr	+	+	high	tr
Oleracein N	-	+	-	-	-	-	-	-	+	high	+	-
Oleracein O	-	-	-	-	-	-	-	-	high	high	high	-
Oleracein K	-	-	-	-	-	-	-	-	high	high	high	-
Oleracein L	-	-	-	-	-	-	-	-	high	high	high	-
Oleracein P	-	-	-	-	-	-	-	-	+	+	high	-
Oleracein Q	-	-	-	-	-	-	-	-	+	+	+	-
Oleracein S	-	-	-	-	+	+	+	-	+	+	+	-

*abundance based on peak height in extracted ion chromatograms and designated: - = not detected; tr = trace $\leqq 1 \times 10^4$; + = moderate $\geqq 1 \times 10^5$ and $\leq 1 \times 10^6$ (+); high $\geqq 1 \times 10^6$.

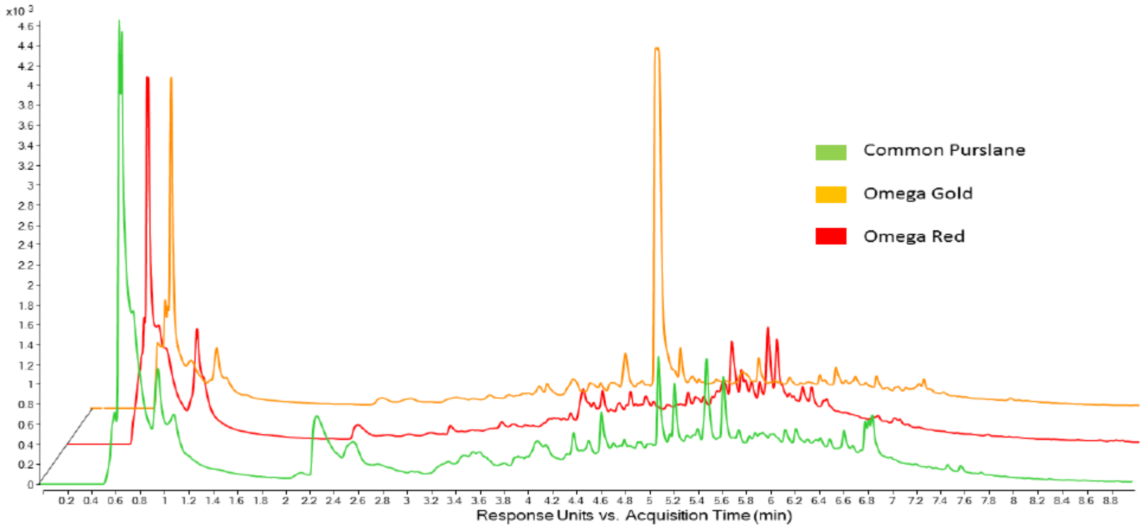

Figure S1. UHPLC-DAD chromatograms generated at 280 nm for leaf extracts of three populations of *Portulaca oleracea*, Common Purslane, Omega Gold, and Omega Red.

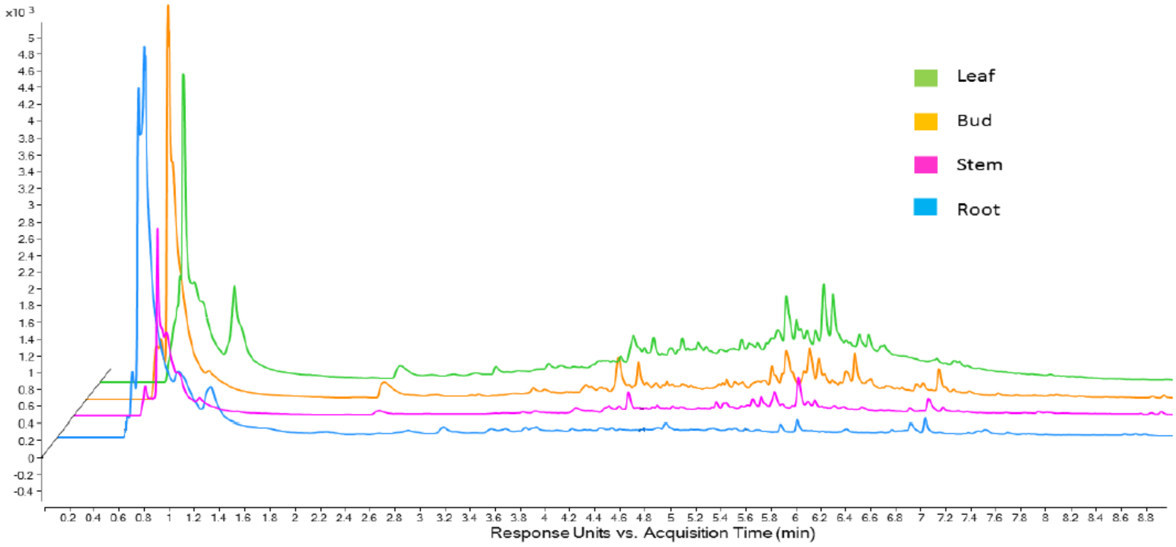

Figure S2. UHPLC-DAD chromatograms generated at 280 nm for extracts of plant parts from *Portulaca oleracea* Omega Red.

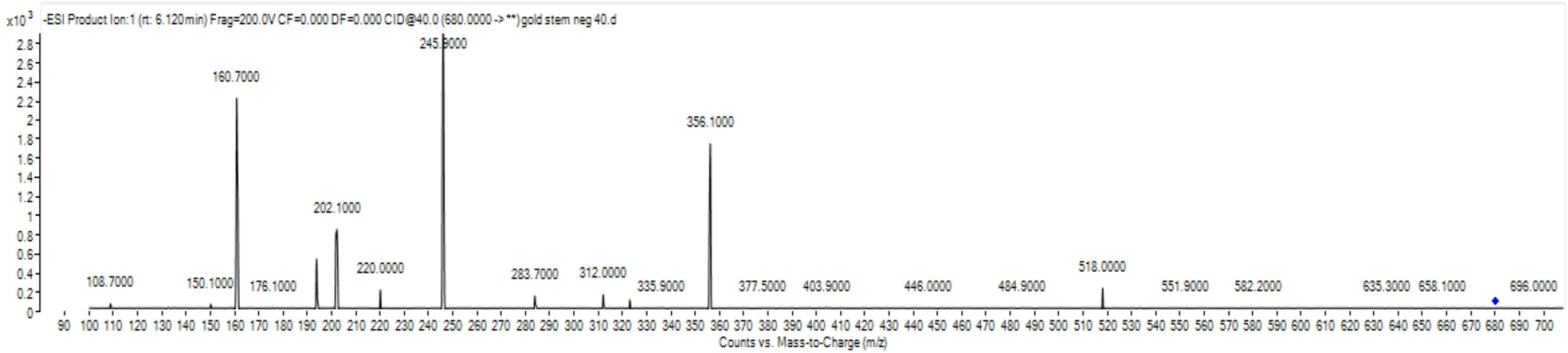

Figure S3. UHPLC-QQQ-MS/MS spectrum derived from the peak at 5.0 min (Figure 4) showing product ions from fragmentation of *m*/*z* 680 [M−H]$^-$. (Retention time 6.12 min is different to that in Figure 4, due to a different instrument being used to produce the two chromatograms).

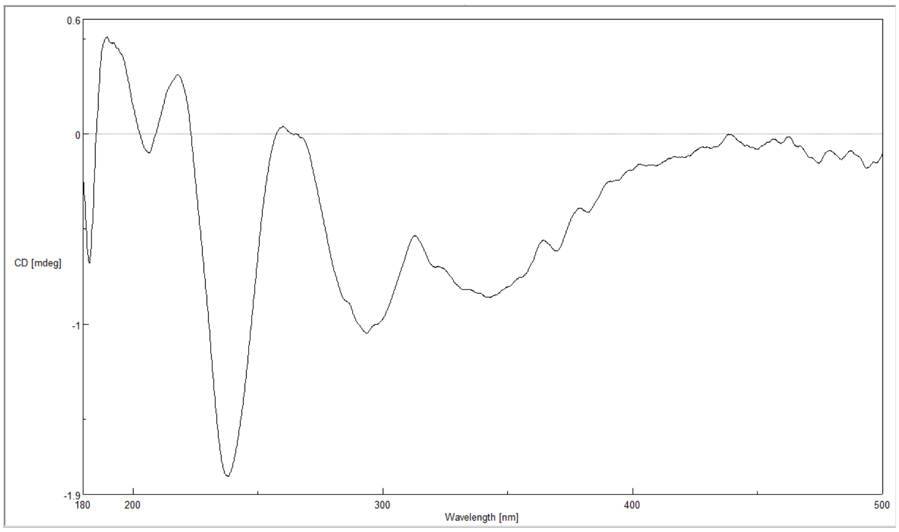

Figure S4a. ECD of Oleracein C

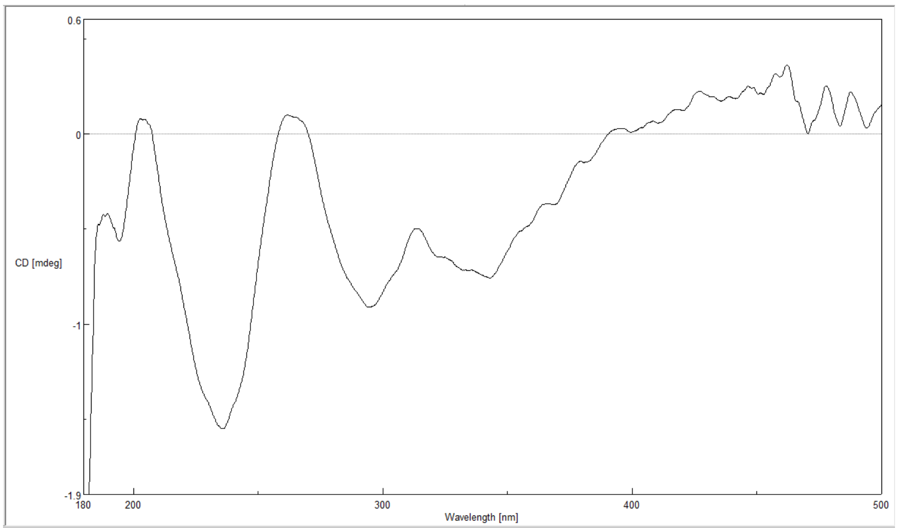

Figure S4b. ECD of oleracein australis 1

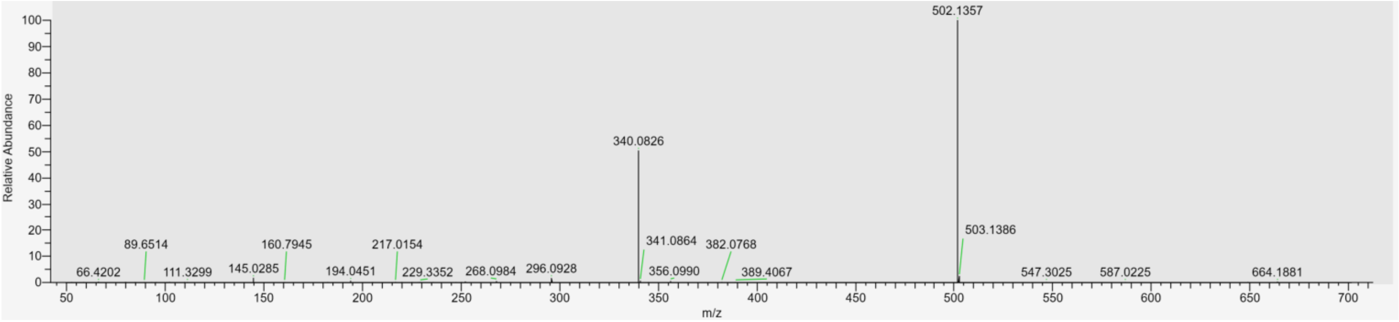

Figure S5a. Negative tandem mass spectrum of oleracein C, collision energy 20 eV (A Q Exactive HF-X Quadrupole-Orbitrap mass-spectrometer (Thermo Fisher Scientific))

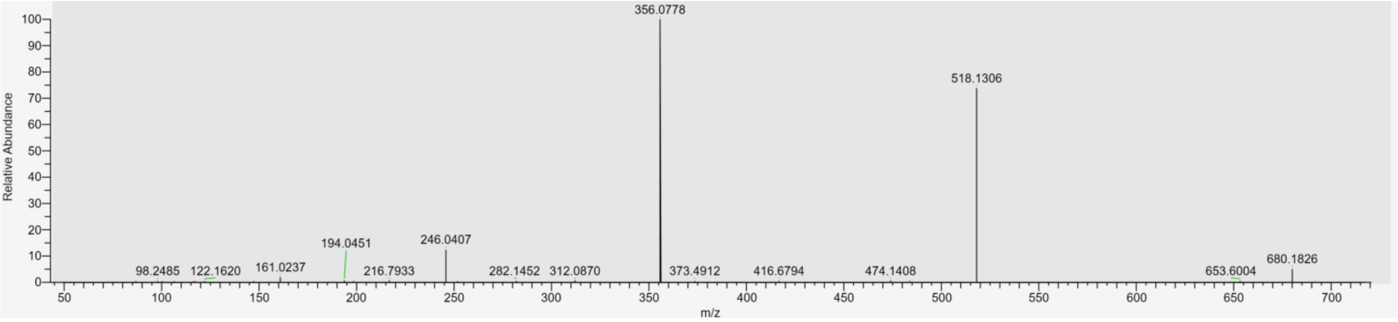

Figure S5b. Negative tandem mass spectrum of oleracein australis 1 collision energy 20 eV (A Q Exactive HF-X Quadrupole-Orbitrap mass-spectrometer (Thermo Fisher Scientific))

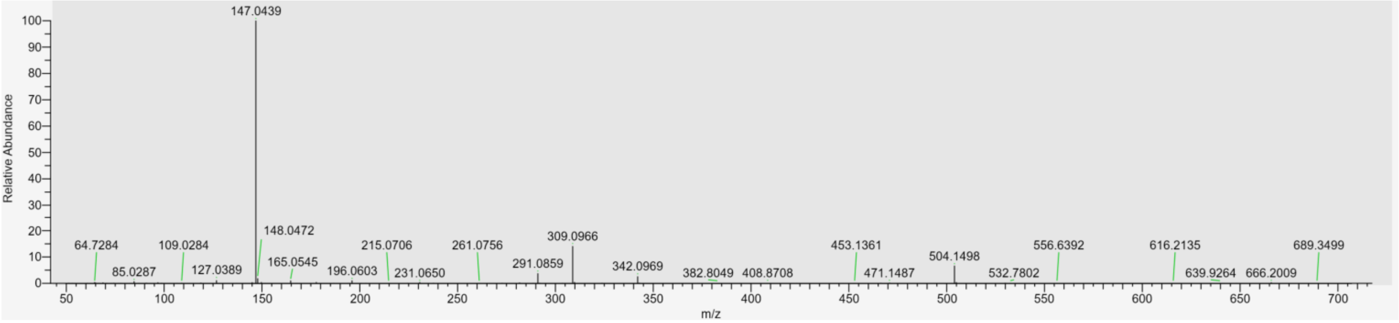

Figure S5c. Positive tandem mass spectrum of oleracein C collision energy 20 eV (A Q Exactive HF-X Quadrupole-Orbitrap mass-spectrometer (Thermo Fisher Scientific))

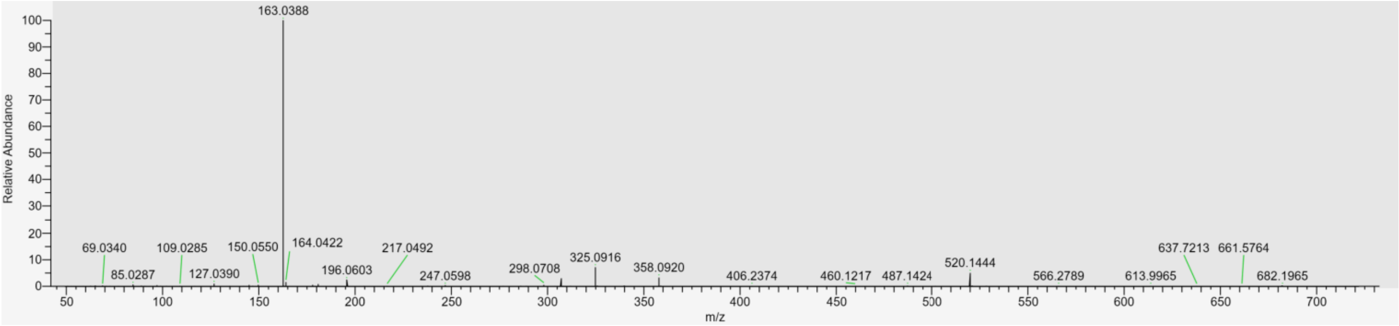

Figure S5d. Positive tandem mass spectrum of oleracein australis 1 collision energy 20 eV (A Q Exactive HF-X Quadrupole-Orbitrap mass-spectrometer (Thermo Fisher Scientific))

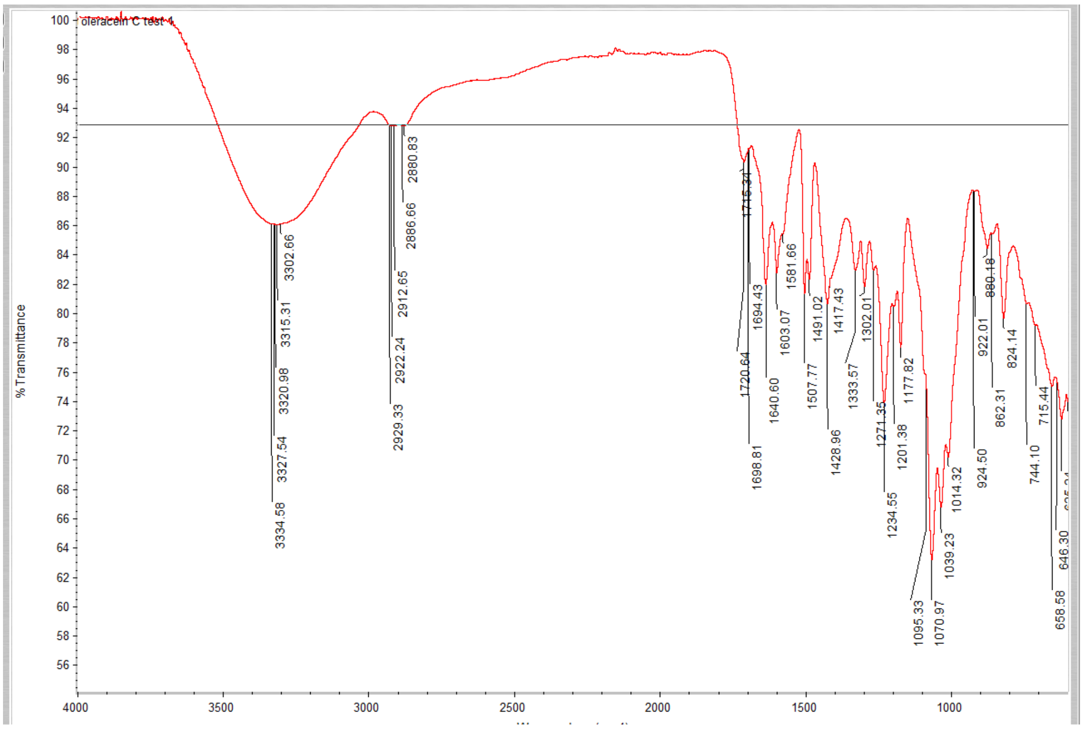

Figure S6. Oleracein C IR spectrum produced using a Nicolet™ iS™5 FT-IR spectrometer (KBr)

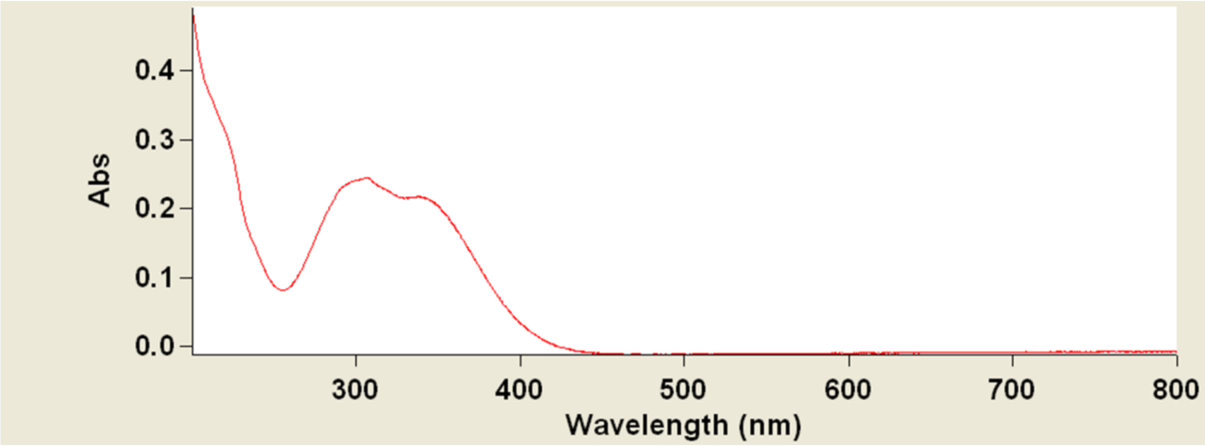

Figure S7. UV-Vis profile of oleracein C produced on a Cary 1 Bio UV-Visible Spectrophotometer

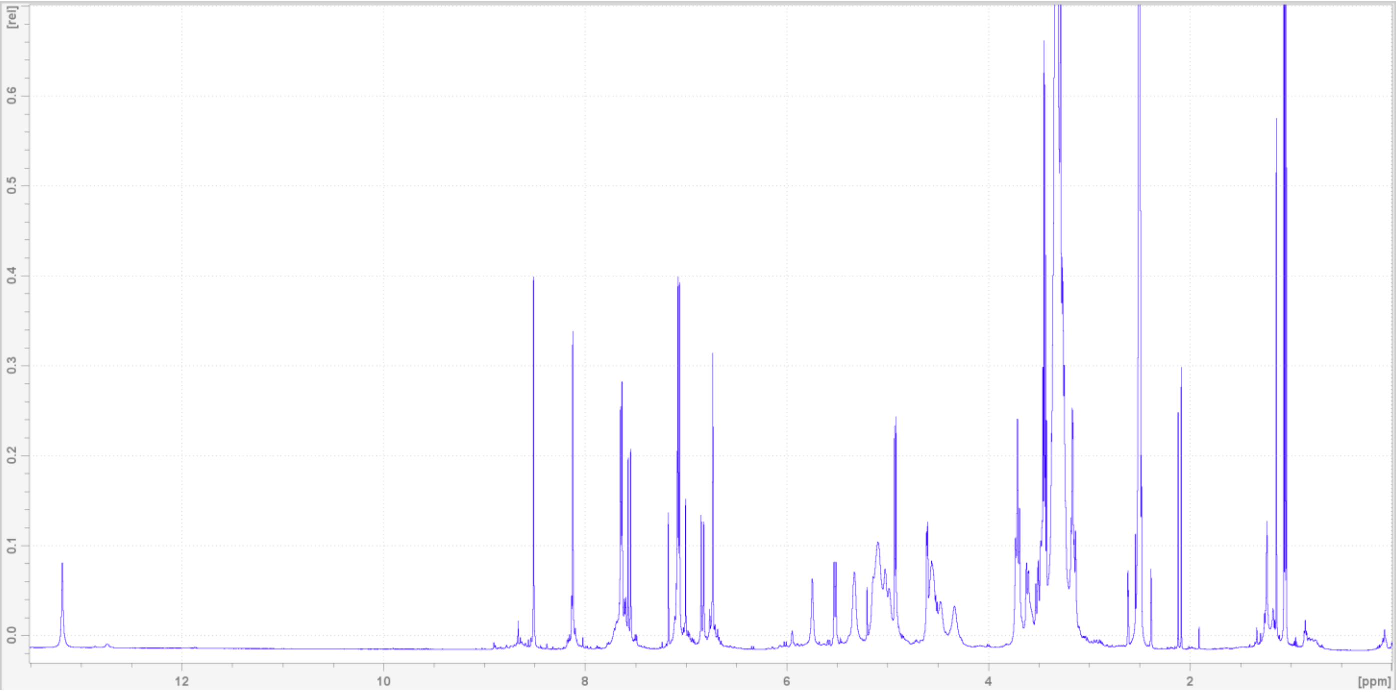

Figure S8a. ^{1}H NMR spectrum of oleracein C in d_6-DMSO produced on Bruker 600‘54 Ascend NMR spectrometer

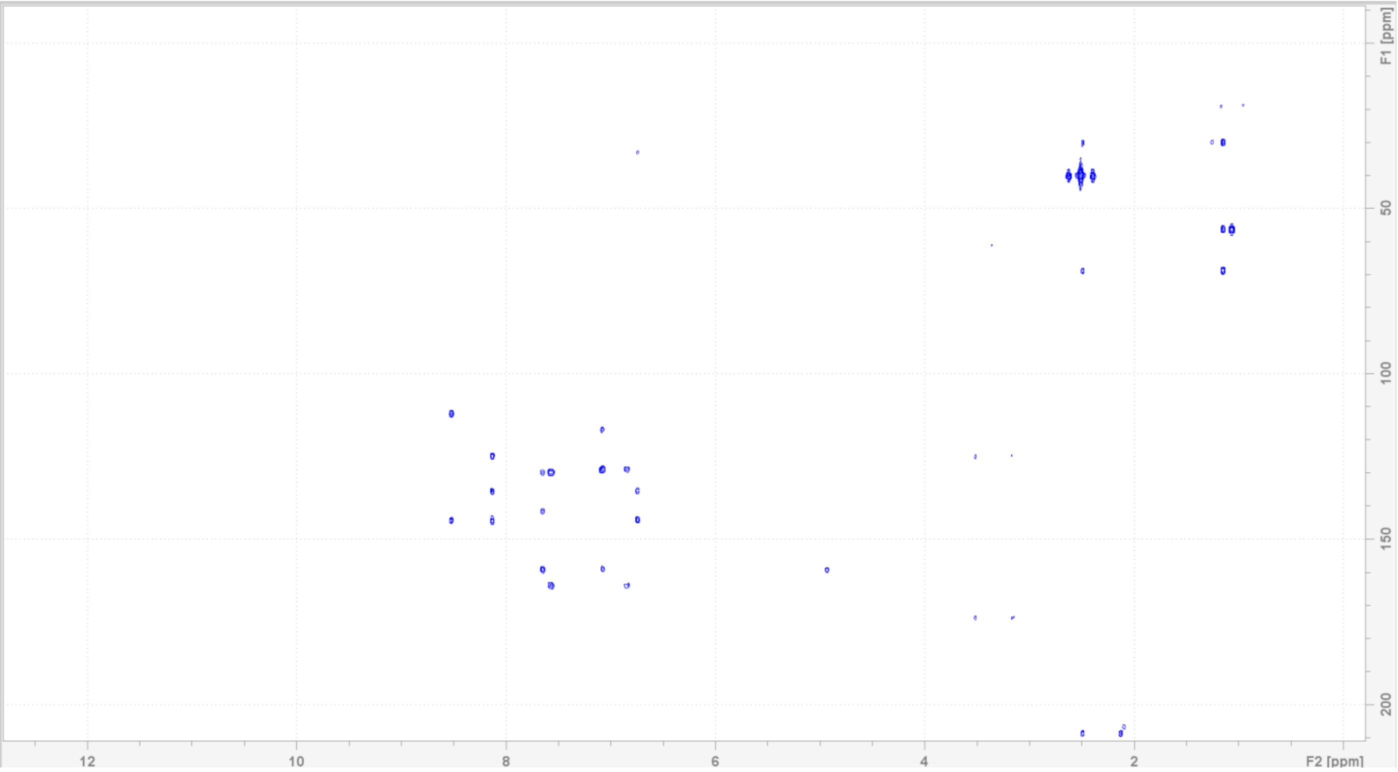

Figure S8b. ^{1}H-^{13}C HMBC NMR spectrum of oleracein C in d_6-DMSO produced on Bruker 600‘54 Ascend NMR spectrometer

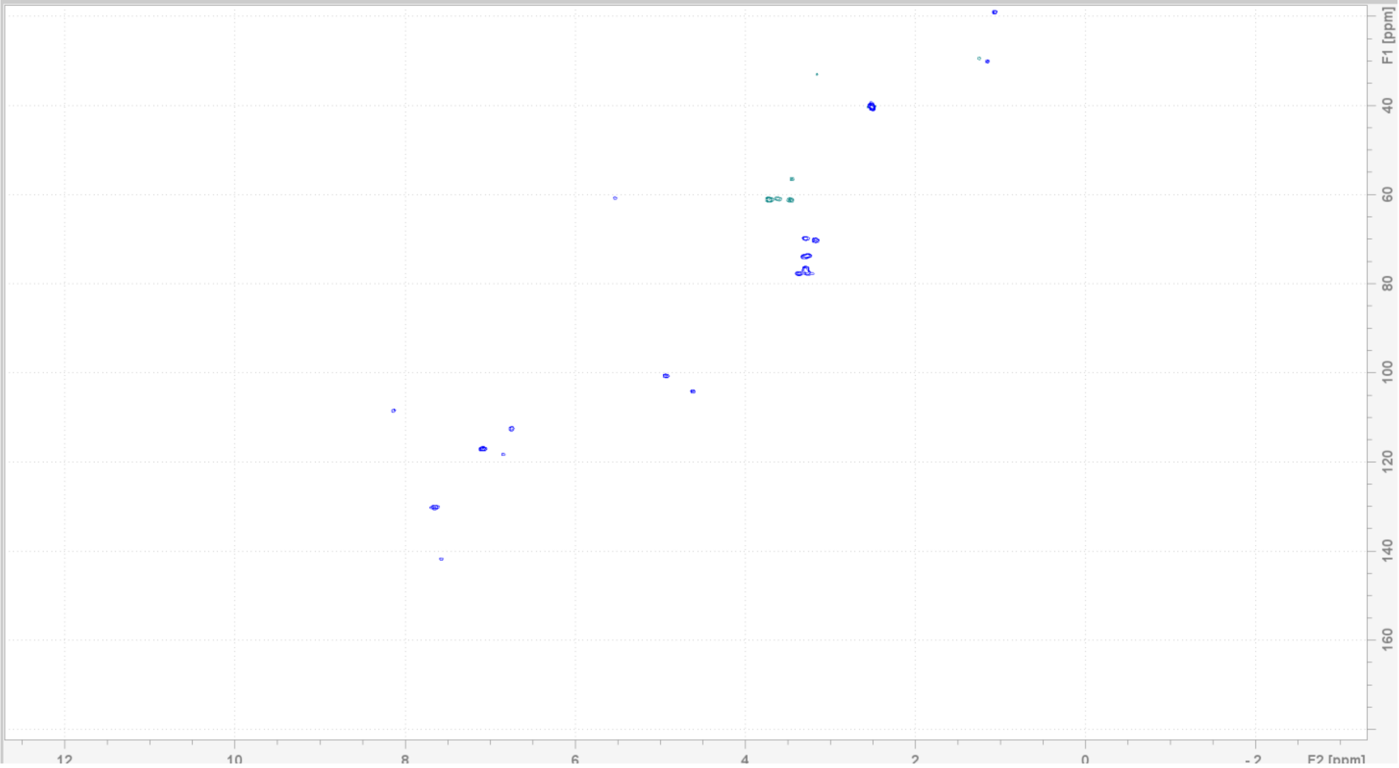

Figure S8c. ^{1}H-^{13}C HSQC NMR spectrum of oleracein C in d_6-DMSO produced on Bruker 600‘54 Ascend NMR spectrometer

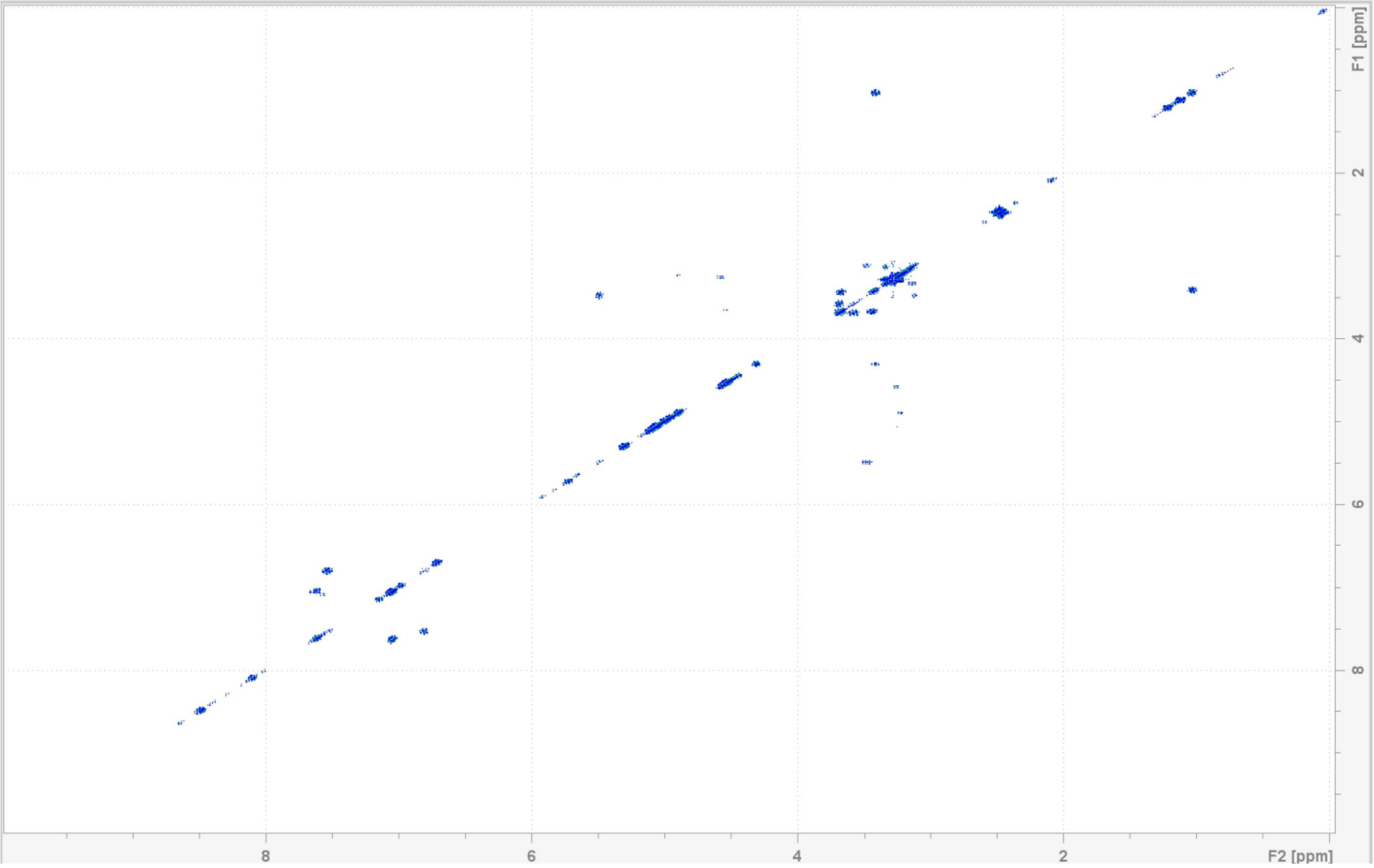

Figure S8d. ^{1}H-^{1}H COSY NMR spectrum of oleracein C in d_6-DMSO produced on Bruker 600‘54 Ascend NMR spectrometer

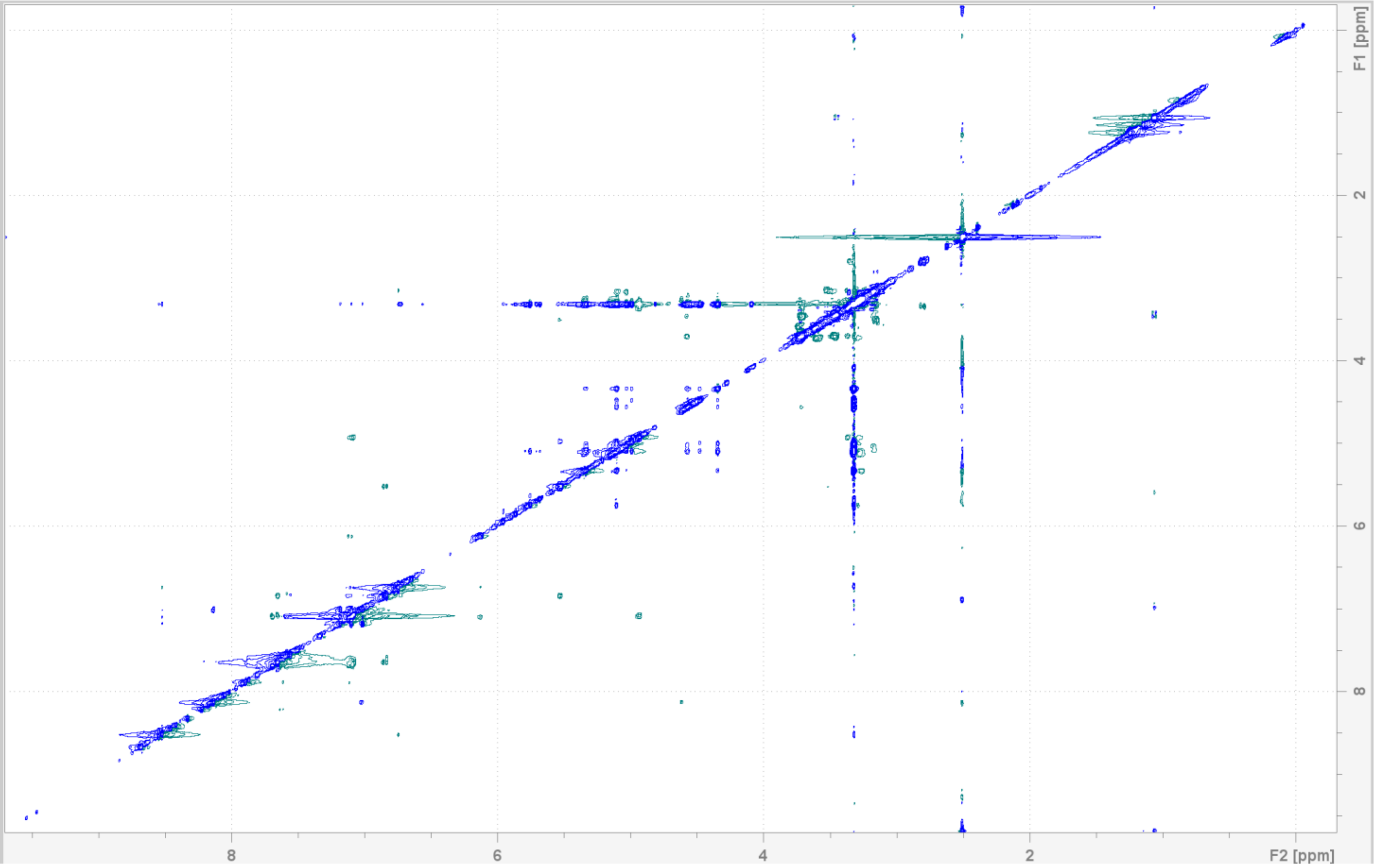

Figure S8e. ^{1}H-^{1}H ROESY NMR spectrum of oleracein C in d_6-DMSO produced on Bruker 600'54 Ascend NMR spectrometer

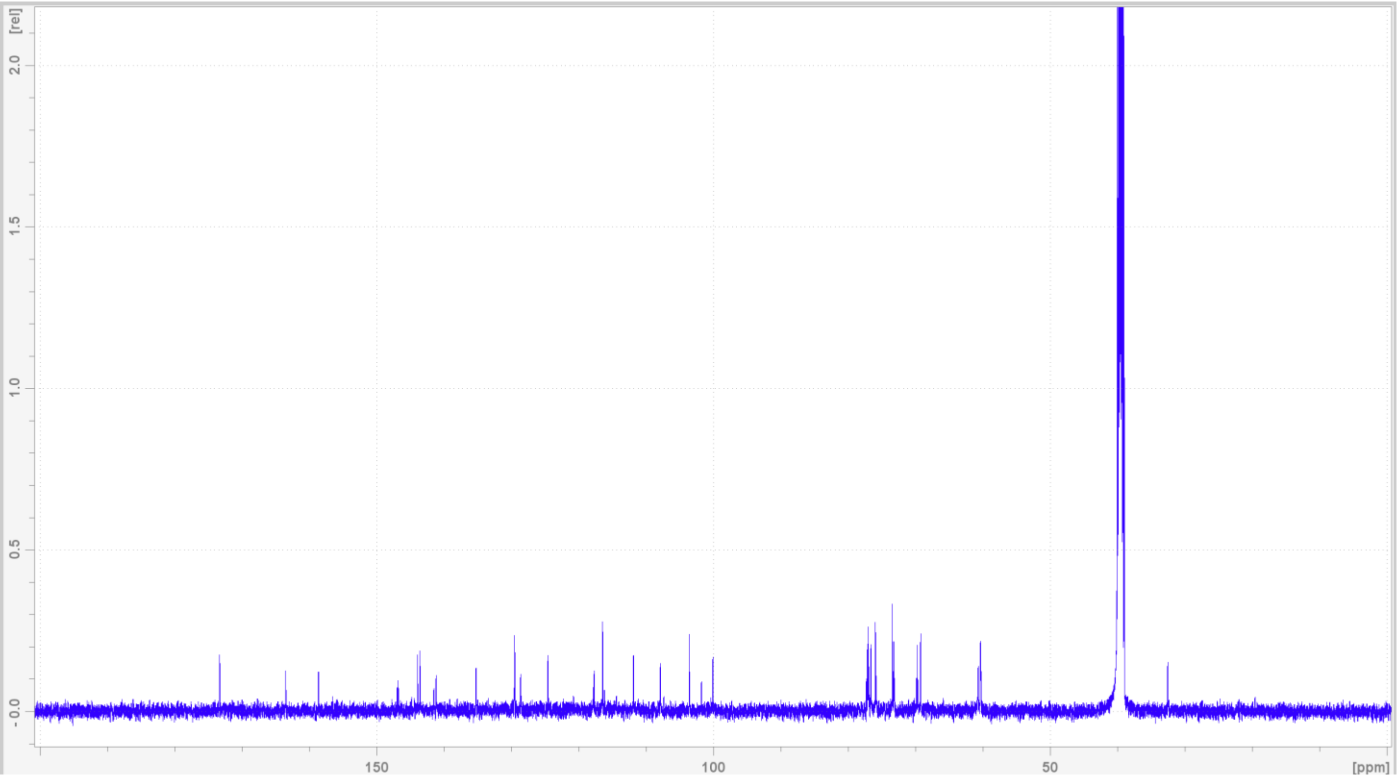

Figure S8f. ^{13}C NMR spectrum of oleracein C in d_6-DMSO produced on Bruker 500'54 Ascend NMR spectrometer

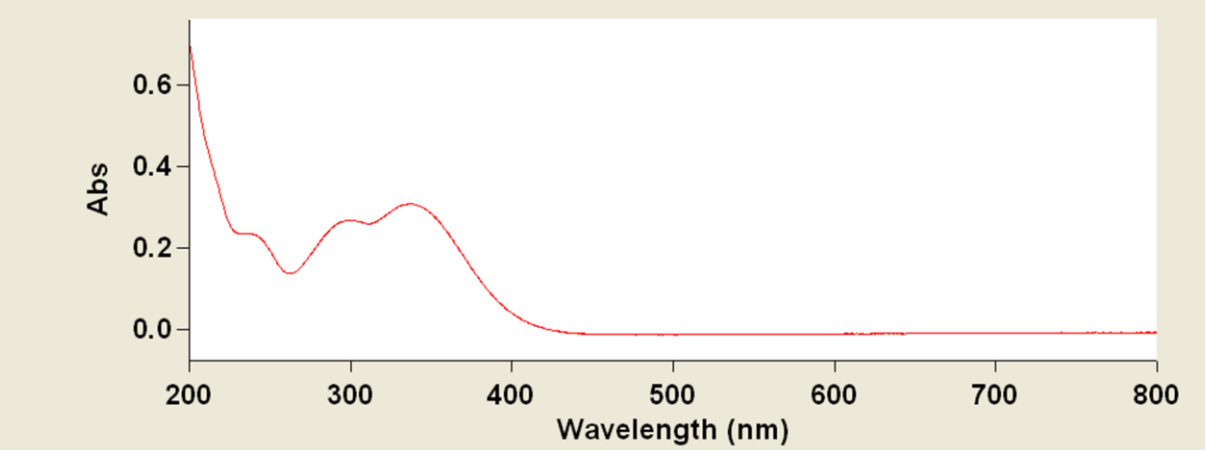

Figure S9. UV-Vis profile of oleracein australis 1 produced on a Cary 1 Bio UV-Visible Spectrophotometer

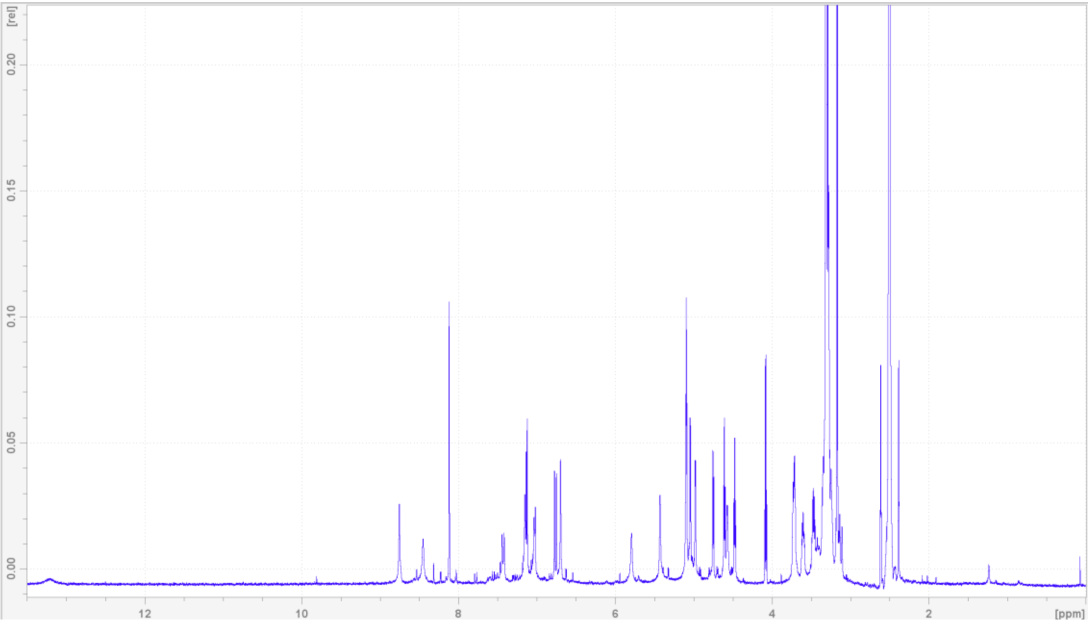

Figure S10a. ^{1}H NMR spectrum of oleracein australis 1 in d_6-DMSO produced on Bruker 600‘54 Ascend NMR spectrometer

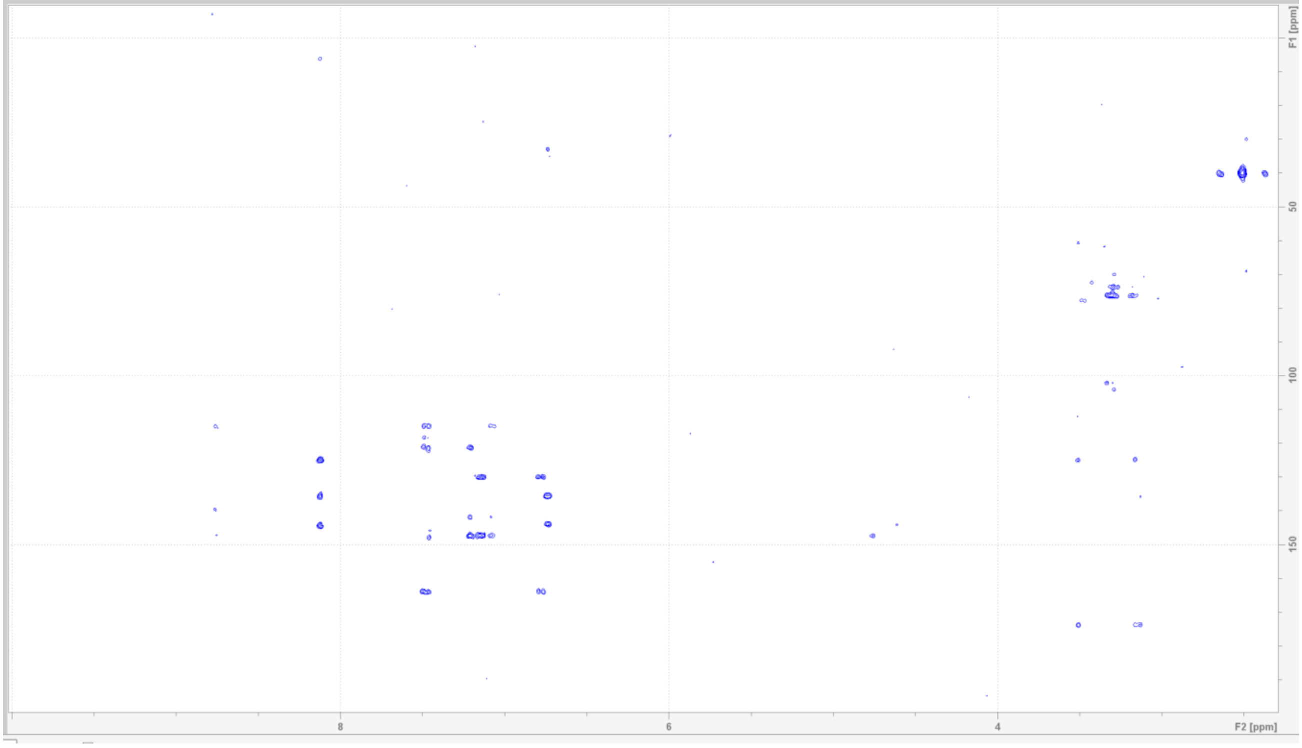

Figure S10b. ^{1}H-^{13}C HMBC NMR spectrum of oleracein australis 1 in d_6-DMSO produced on Bruker 600‘54 Ascend NMR spectrometer

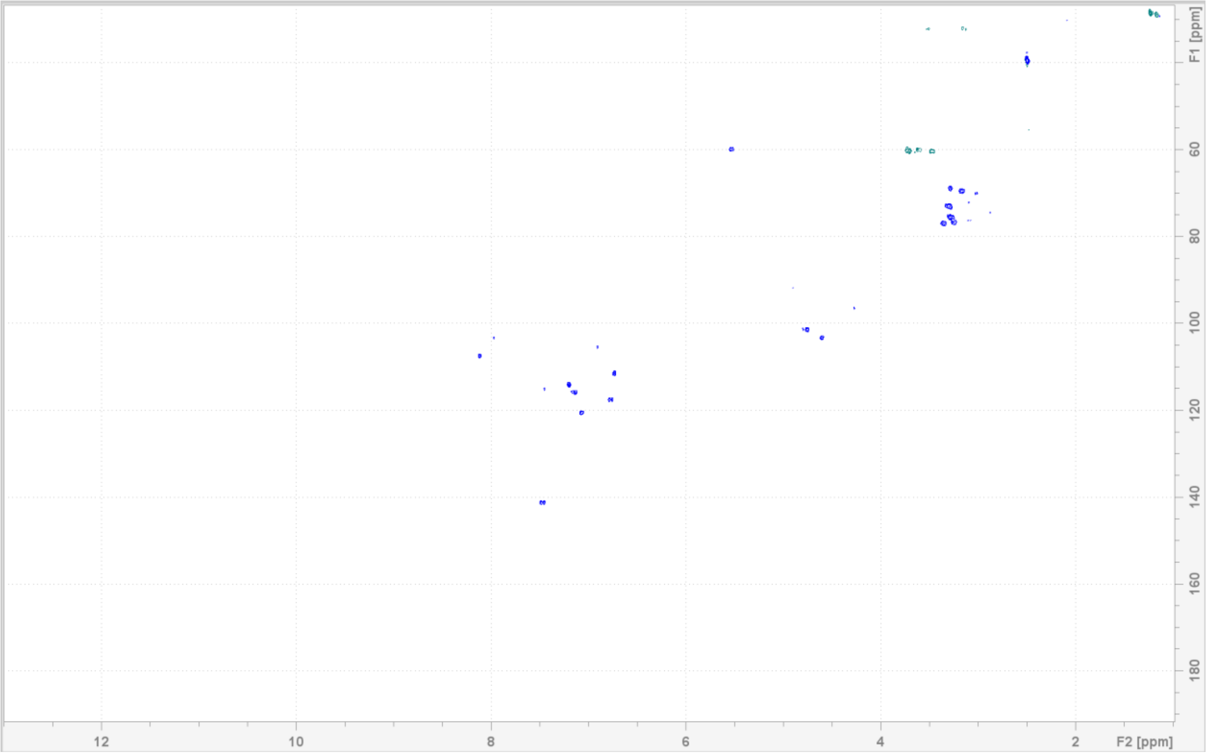

Figure S10c. ^{1}H-^{13}C HSQC NMR spectrum of oleracein australis 1 in d_6-DMSO produced on Bruker 600‘54 Ascend NMR spectrometer

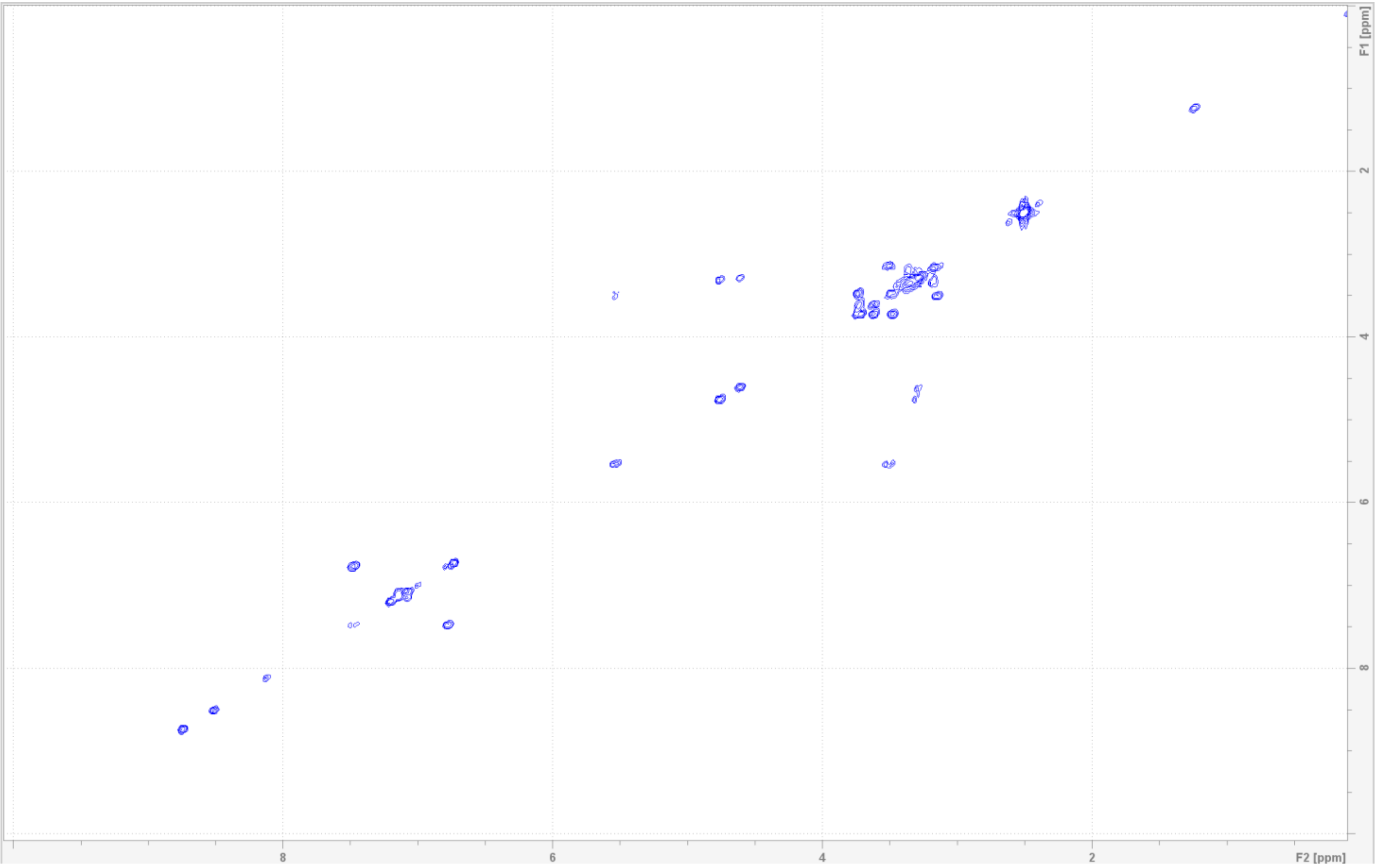

Figure S10d. ^{1}H-^{1}H COSY NMR spectrum of oleracein australis 1 in d_6-DMSO produced on Bruker 600‘54 Ascend NMR spectrometer

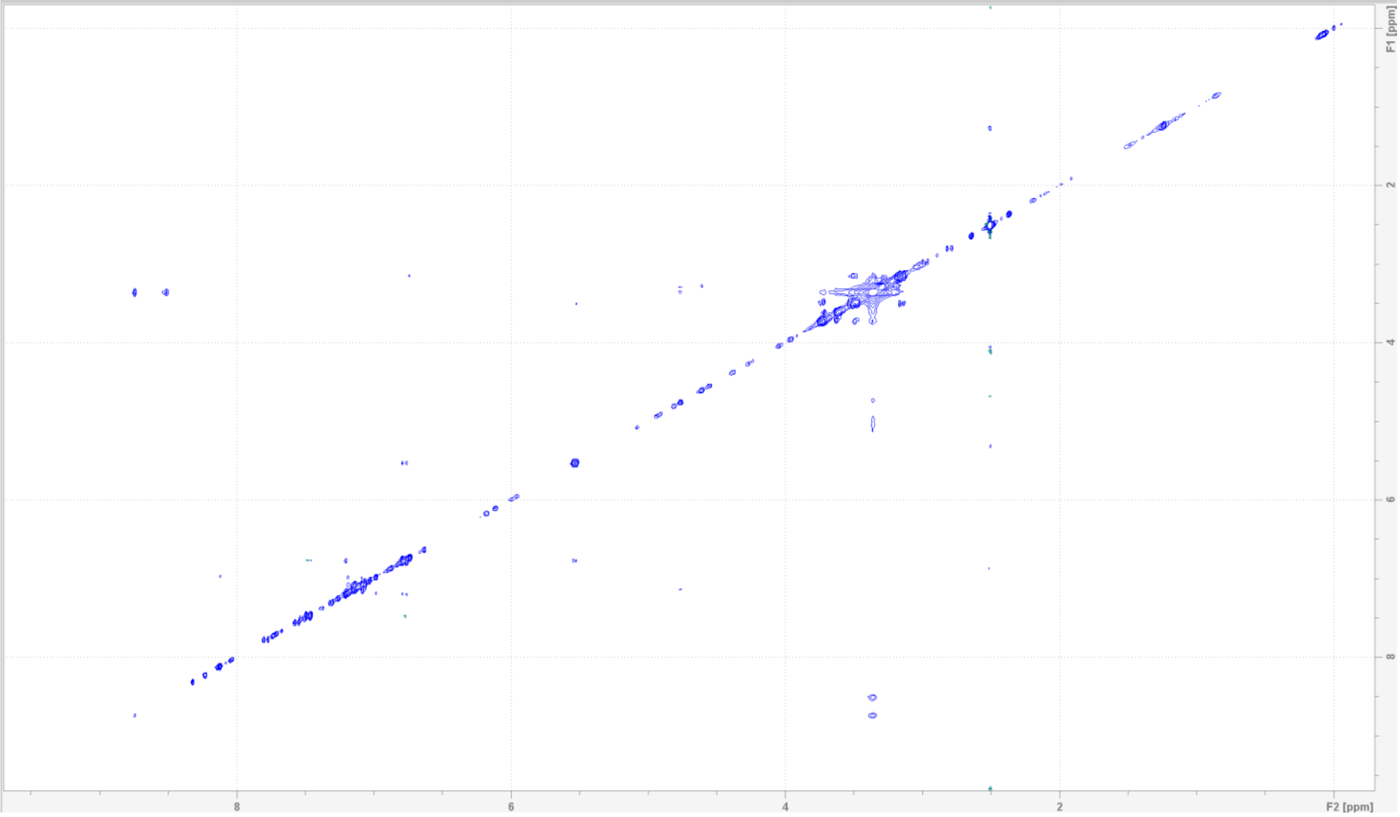

Figure S10e. ^{1}H-^{1}H ROESY NMR spectrum of oleracein australis 1 in d_6-DMSO produced on Bruker 600'54 Ascend NMR spectrometer

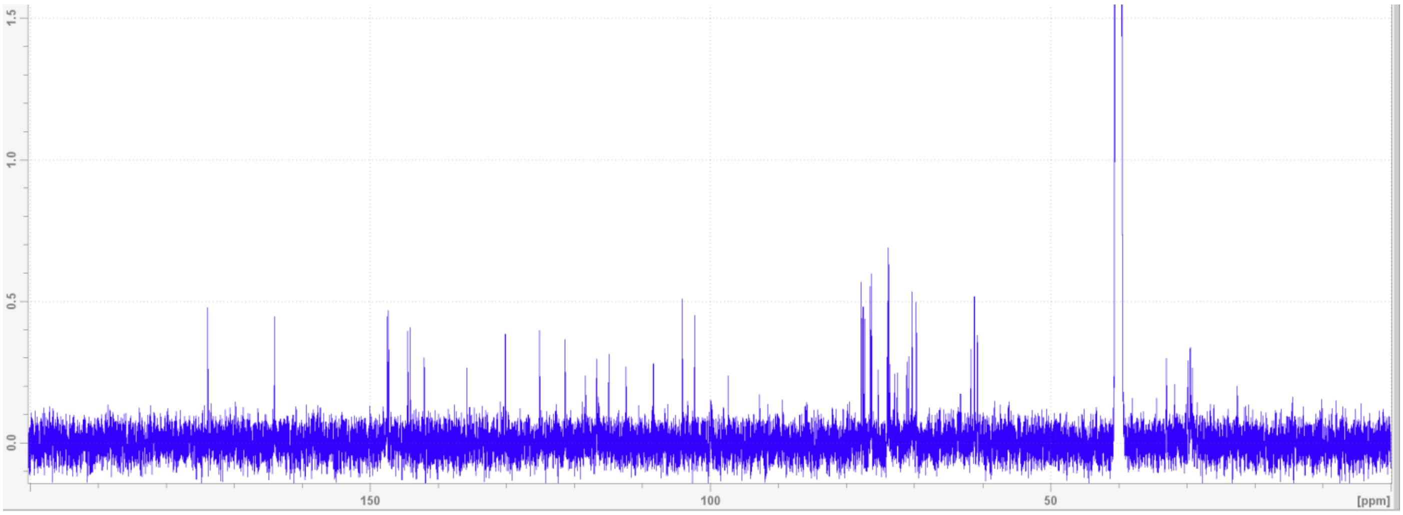

Figure S10f. ^{13}C NMR spectrum of oleracein australis 1 in d_6-DMSO produced on Bruker 500'54 Ascend NMR spectrometer

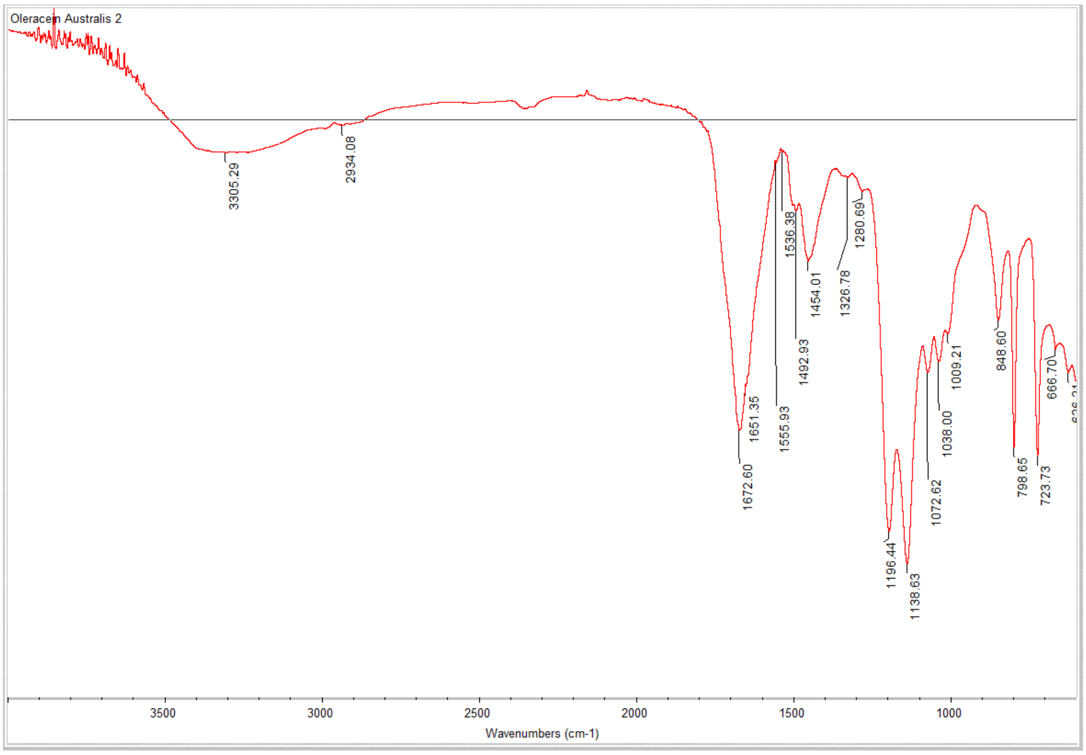

Figure S11. Oleracein australis 1 IR spectrum produced using a Nicolet™ iS™5 FT-IR spectrometer (KBr)

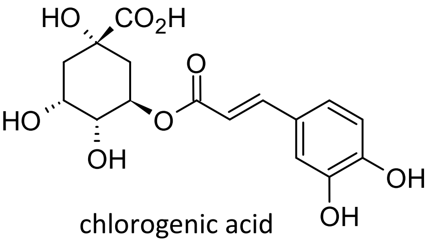
chlorogenic acid

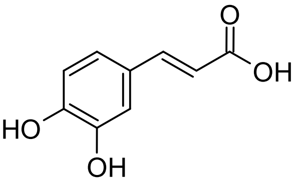
caffeic acid

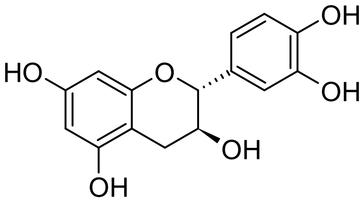
catechin

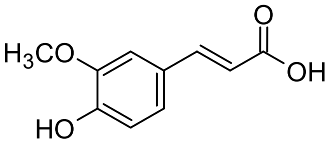
ferulic acid

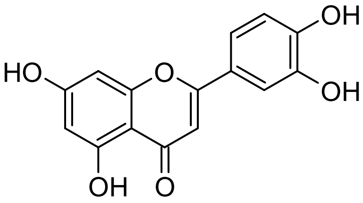
luteolin

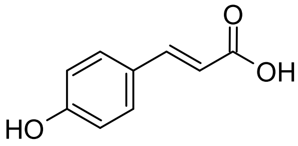
p-coumaric acid

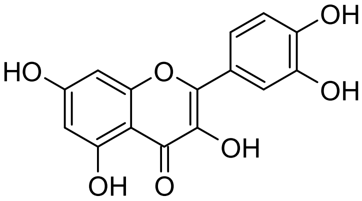
quercetin

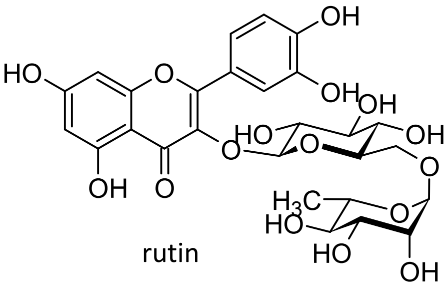
rutin

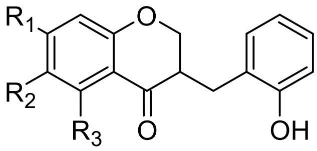

R_1=OMe, R_2=H, R_3=OMe portulacanone A

R_1=OMe, R_2=OMe, R_3=OMe portulacanone B

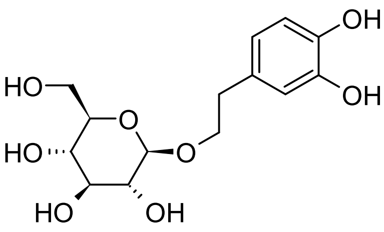
hydroxytyrosol 1-*O*-glucoside

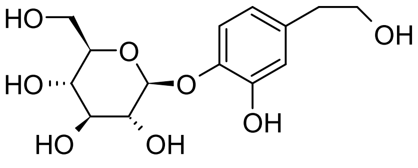
hydroxytyrosol 4-*O*-glucoside

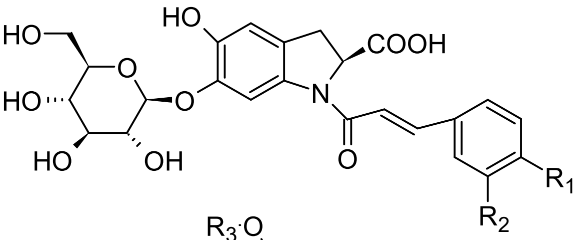

oleracein A	R_1 = OH	R_2 = H
oleracein B	R_1 = OH	R_2 = CH_3O
oleracein C	R_1 = O-Glc	R_2 = H
oleracein D	R_1 = O-Glc	R_2 = CH_3O

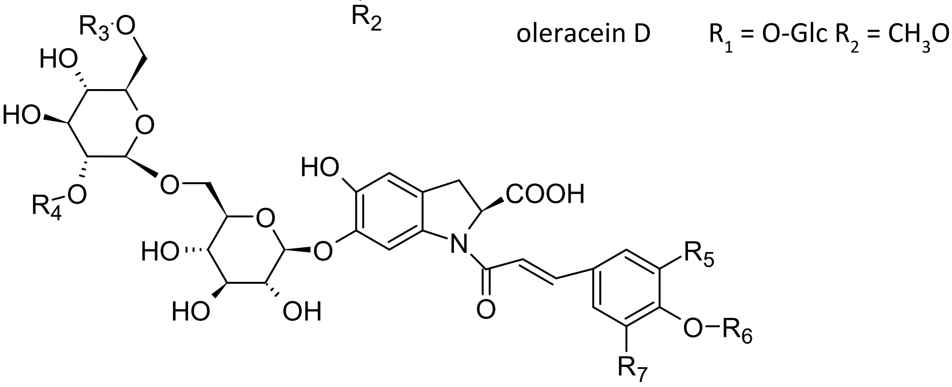

oleracein H	R_3 = H	R_4 = H	R_5 = H	R_6 = H	R_7 = H
oleracein I	R_3 = H	R_4 = H	R_5 = H	R_6 = H	R_7 = CH_3O
oleracein J	R_3 = Fer	R_4 = H	R_5 = OH	R_6 = H	R_7 = H
oleracein K	R_3 = H	R_4 = Caf	R_5 = H	R_6 = H	R_7 = H
oleracein L	R_3 = H	R_4 = Caf	R_5 = CH_3O	R_6 = H	R_7 = H
oleracein N	R_3 = H	R_4 = Fer	R_5 = H	R_6 = H	R_7 = H
oleracein O	R_3 = H	R_4 = Fer	R_5 = H	R_6 = H	R_7 = CH_3O
oleracein P	R_3 = H	R_4 = H	R_5 = H	R_6 = Glc	R_7 = H
oleracein Q	R_3 = H	R_4 = H	R_5 = CH_3O	R_6 = Glc	R_7 = H
oleracein S	R_3 = Fer	R_4 = H	R_5 = H	R_6 = H	R_7 = H
oleracein X	R_3 = H	R_4 = H	R_5 = OH	R_6 = H	R_7 = H

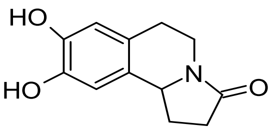

oleracein [illegible]

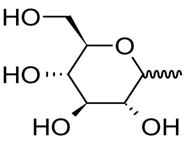

glucosyl [illegible]

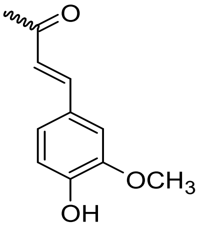

feruloyl [illegible]

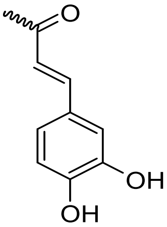

caffeoyl [illegible]

Figure S12. Structures of compounds reported in Australian populations of *Portulaca oleracea*.
